# Uncovering Structure–Activity Relationships in Pt/CeO_2_ Catalysts for Hydrogen-Borrowing Amination

**DOI:** 10.1021/acscatal.2c04347

**Published:** 2023-01-05

**Authors:** Tao Tong, Mark Douthwaite, Lu Chen, Rebecca Engel, Matthew B. Conway, Wanjun Guo, Xin-Ping Wu, Xue-Qing Gong, Yanqin Wang, David J. Morgan, Thomas Davies, Christopher J. Kiely, Liwei Chen, Xi Liu, Graham J. Hutchings

**Affiliations:** † Cardiff Catalysis Institute, School of Chemistry, Cardiff University, Main Building, Park Place, CardiffCF10 3AT, U.K.; ‡ Key Laboratory for Advanced Materials and Joint International Research Laboratory of Precision Chemistry and Molecular Engineering, Feringa Nobel Prize Scientist Joint Research Center, Research Institute of Industrial Catalysis, School of Chemistry and Molecular Engineering, East China University of Science and Technology, Shanghai200237, China; § Department of Materials Science and Engineering, Lehigh University, 5 East Packer Avenue, Bethlehem, Pennsylvania18015, United States; ∥ School of Chemistry and Chemical, In-situ Centre for Physical Sciences, Frontiers Science Centre for Transformative Molecules, Shanghai Jiao Tong University, 200240Shanghai, P. R. China

**Keywords:** Pt/CeO_2_, hydrogen borrowing, transfer hydrogenation, green chemistry, amination

## Abstract

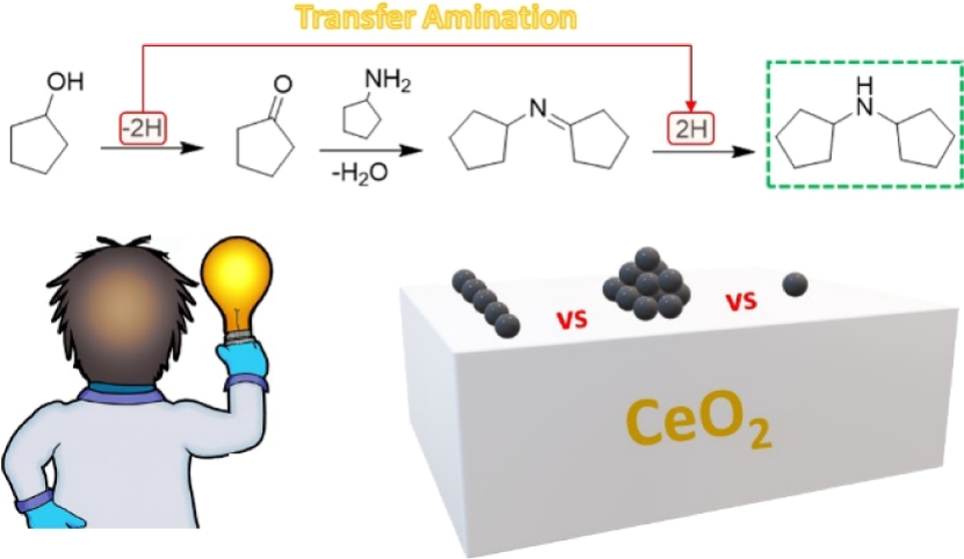

The hydrogen-borrowing amination of alcohols is a promising route to produce amines. In this study, experimental parameters involved in the preparation of Pt/CeO_2_ catalysts were varied to assess how physicochemical properties influence their performance in such reactions. An amination reaction between cyclopentanol and cyclopentylamine was used as the model reaction for this study. The Pt precursor used in the catalyst synthesis and the properties of the CeO_2_ support were both found to strongly influence catalytic performance. Aberration corrected scanning transmission electron microscopy revealed that the most active catalyst comprised linearly structured Pt species. The formation of these features, a function result of epitaxial Pt deposition along the CeO_2_ [100] plane, appeared to be dependent on the properties of the CeO_2_ support and the Pt precursor used. Density functional theory calculations subsequently confirmed that these sites were more effective for cyclopentanol dehydrogenation—considered to be the rate-determining step of the process—than Pt clusters and nanoparticles. This study provides insights into the desirable catalytic properties required for hydrogen-borrowing amination but has relevance to other related fields. We consider that this study will provide a foundation for further study in this atom-efficient area of chemistry.

## 1 Introduction

Amines have important roles in many aspects of the modern industry, including the production of dyes, polymers, pharmaceutical/agrochemical reagents, and other value-added chemicals.^1−3^ Many conventional strategies for the synthesis of organic amines have been well-established for Hofmann alkylation,^4^ nucleophilic addition,^5^ hydrogenation of nitriles and nitroarenes,^6,7^ hydroamination,^8^ and others. However, these established methods are often limited by poor selectivity, require the use of environmentally damaging chemicals, produce highly toxic byproducts, and typically employ harsh reaction conditions.^9−11^ With stricter environmental legislation continually being imposed, the development of novel sustainable strategies for the economic production of amines must be considered.

Recently, some progress in the development of clean, sustainable amination methods has been achieved. Two notable strategies for this are hydrogen-borrowing amination and reductive amination.^12−18^ Compared with the reductive amination route from aldehydes or ketones, hydrogen-borrowing amination from alcohols does not require a hydrogen overpressure as the hydrogen required for the process stems from the dehydrogenation of the alcohol. This makes the process far more atom efficient and sustainable while reducing overhead costs from hydrogen consumption, which is almost exclusively derived from fossil fuel feedstocks.^18,19^ Furthermore, industrial sources of alcohol compounds are more sufficient than those of aldehydes/ketones.^20^ Thus, the hydrogen-borrowing amination strategy provides many advantages for industrial scale-up.

In a typical hydrogen-borrowing amination process, using alcohols as the substrate, the alcohol is first dehydrogenated to form the corresponding aldehyde/ketone, which is proposed to be the rate-determining step (RDS) of the process.^21,22^ Following this, the aldehyde/ketone undergoes condensation with the selected amine/ammonia to form the corresponding imine/Schiff base intermediate, with water formed as a byproduct. Finally, the N-containing intermediate is hydrogenated with the hydrogen species generated in the initial dehydrogenation.^23^

Pt-based heterogeneous catalysts are predicted to be highly active for oxidant-free alcohol dehydrogenation^24^ and practically exhibit a remarkable catalytic activity for a range of dehydrogenation reactions.^25,26^ These features indicate the potential of using Pt catalysts for hydrogen-borrowing reactions. Recent research also confirmed that CeO_2_ supports—due to their reducible nature—are highly effective for metal-loaded catalysts in hydrogen-borrowing amination.^18,27^ It was, therefore, of interest to conduct an in-depth investigation on the use of Pt/CeO_2_ catalysts for hydrogen-borrowing amination to gain further insights into the desirable catalytic properties required for this emerging field of chemistry.

**Scheme 1:**
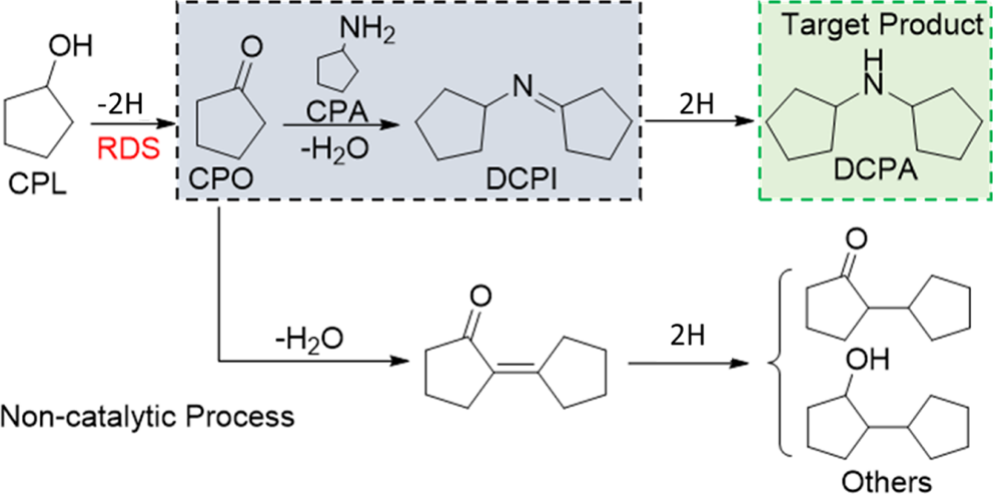
Reaction Network of Hydrogen-Borrowing Amination over Pt/CeO_2_ Catalysts; Keys: CPL (Cyclopentanol); CPA (Cyclopentylamine); DCPA (Dicyclopentylamine); DCPI (*N-cyclopentylcyclopentanamine*); CPO (Cyclopentanone); and Others {[1,1′-Bi(cyclopentan)]-2-one and [1,1′-Bi(cyclopentan)]-2-ol}

Herein, we focus on the design and synthesis of Pt/CeO_2_ catalysts for an amination reaction. We have selected an amination reaction between cyclopentanol (CPL) and cyclopentylamine (CPA) (Scheme 1) as our model reaction to establish structure–activity relationships. Through combining catalytic performance tests with comprehensive characterization, we find that the structure of Pt species over ceria supports has a significant impact on the observed catalytic performance in the reaction. Of the catalysts assessed, a Pt/CeO_2_ catalyst possessing Pt atoms that exist in linear conformations [either as two-dimensional (2D) rafts or as one-dimensional rows of Pt atoms] exhibited the best performance. The formation of these unique Pt species was attributed to epitaxial Pt deposition, and as such, the physicochemical properties of the support have a significant impact on the reactivity of the catalyst. These findings were supported by density functional theory (DFT) calculations. Furthermore, we found that the Pt precursor and reductive conditions used significantly influenced catalyst performance.

## 2 Experimental Section

### 2.1 Chemicals (Source and Purity)

Cerium (III) nitrate hexahydrate (Sigma-Aldrich, 99%); CPL (Sigma-Aldrich, 99%); CPA (Sigma-Aldrich, 99%); decane (Sigma-Aldrich, anhydrous, ≥99%); hexachloroplatinic acid hydrate (Sigma-Aldrich, ∼38% Pt basis); *p*-xylene (Sigma-Aldrich, anhydrous, ≥99%); tetraammineplatinum (II) nitrate (Sigma-Aldrich, 99.995%); and additional commercial CeO_2_ materials were also acquired from Alfa Aesar (CeO_2_-AA) and Sigma-Aldrich (CeO_2_-Sigma). We also synthesized an additional CeO_2_ support in-house from the thermal decomposition of cerium (III) nitrate hexahydrate (CeO_2_-DE).

### 2.2 Catalyst Preparation

In this study, we prepared our 1% Pt/CeO_2_ catalysts using the three CeO_2_ supports discussed in Section 2.1: CeO_2_-AA, CeO_2_-Sigma, and CeO_2_-DE. The in-house CeO_2_-DE material was obtained by calcining Ce(NO_3_)_3_·6H_2_O (4 g) in air at 500 °C for 4 h (ramp rate: 10 °C·min^–1^). The specific surface area of each CeO_2_ support was determined using the Brunauer–Emmett–Teller (BET) method, the results from which are listed in Table 1. Experimental protocols for conducting these measurements are listed in Section 2.4.

**Table 1:** Specific Areas of Three CeO_2_ Supports Were Determined by the BET Method, Using a Five-Point Plot between *p*/*p*_0_ Values of 0.05–0.3 and Using N_2_ as the Sorbate

Support ID	Specific Area (m^2^/g)
CeO_2_-DE	103
CeO_2_-AA	73
CeO_2_-Sigma	43

Two Pt precursors, H_2_PtCl_6_ and (NH_3_)_4_Pt(NO_3_)_2_, were subsequently used to impregnate Pt onto the different supports. For this, aqueous solutions of H_2_PtCl_6_ (3.319 g/L) and (NH_3_)_4_Pt(NO_3_)_2_ (2.482 g/L) were first prepared.

Pt/CeO_2_ catalysts prepared using H_2_PtCl_6_ are denoted as 1% Pt/CeO_2_-H. A typical experimental procedure used to synthesize such a catalyst is as follows: CeO_2_ (1 g) was added to an aqueous solution of H_2_PtCl_6_ (8 mL, 3.319 g/L). The mixture was subsequently stirred at room temperature for 2 h, before being heated to 60 °C, where it was held with stirring for 12 h; this meant that residual water present in the mixture evaporated slowly. To ensure complete removal of water was achieved, the catalyst was dried for a further 12 h in an oven at 110 °C. Before use, the catalysts were reduced under a flow of 5% H_2_/Ar (100 mL·min^–1^) for 2 h at a stated temperature (150–350 °C) using a ramp rate of 5 °C·min^–1^.

A similar process was adapted to synthesize the samples using (NH_3_)_4_Pt(NO_3_)_2_ aqueous solution, which is denoted as 1% Pt/CeO_2_-N. In short, CeO_2_ support (1 g) was added into an aqueous solution of (NH_3_)_4_Pt(NO_3_)_2_ (8 mL, 2.482 g/L). The mixture was vigorously stirred at room temperature for 2 h. Then, the mixture was heated to 60 °C and kept at this temperature for 12 h to evaporate the water from the system. After that, the sample was further dried in an oven at 110 °C for another 12 h. The obtained solid was calcined under static air at 500 °C for 2 h (ramp rate: 10 °C·min^–1^) before being reduced at a stated temperature (150–350 °C) for 2 h under a flow of 5% H_2_/Ar (ramp rate: 5 °C·min^–1^). The postimpregnation calcination step was only employed for the preparation of the series of Pt/CeO_2_-N catalysts to ensure that a complete decomposition of the nitrate precursor had been achieved. This was, therefore, not required for the preparation of the analogous Pt/CeO_2_-H series. Please note that reductive heat treatments were conducted immediately before any testing or characterization of the catalysts was conducted. The metal loading of all the synthesized catalysts was determined by inductively coupled plasma–mass spectrometry (ICP–MS) and is listed in Table S1.

The catalysts prepared by the aforementioned procedures are denoted as 1% Pt/CeO_2_-X-Y-Z, where X indicates the source of the CeO_2_ (Sigma, AA or DE); Y indicates the Pt precursor used, either H_2_PtCl_6_ (H) or (NH_3_)_4_Pt(NO_3_)_2_ (N); and Z indicates the reduction temperature used (150–350 °C).

### 2.3 Catalytic Activity Experiments

Hydrogen-borrowing amination reactions were conducted in a glass Colaver reactor (50 mL). In a typical reaction, CPL (2 mmol), cyclopentenyl amine (2 mmol), decane (1.54 mmol), the catalyst (50 mg), and *p*-xylene (81.110 mmol) were first added to the reactor. Decane and *p*-xylene were used as the internal standard (Int.S) and solvent in each reaction, respectively. The reactor was then purged with N_2_ five times (conditions: 2 bar, 15 s each time). After the purging procedure, the reactor was pressurized with N_2_ (2 bar). Then, the Colavor reactor was placed in an oil bath, which was set to the predetermined temperature (140 °C). After the reaction was finished, the reactor was cooled in an ice-water bath to quench the reaction before depressurizing. The catalyst was then separated from the reaction effluent by centrifugation and analyzed by gas chromatography (GC) (Aglient 7820A, equipped with an HP-5 column and an FID). Quantification of the reaction products was conducted by comparing GC traces with external calibrations. Qualitative identification of products in the reaction was determined by GC–MS (Shimadzu GCMS-QP2010SE) using the HP-5 column.

The conversion of CPL and CPA is calculated using the following formula
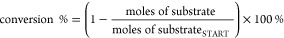
1Here, the same formula is used to calculate the conversion of both CPL and CPA in the reactions. Note that “moles of substrate_START_” corresponds to the moles of the given substrate at the start of the reaction.

The yield of each individual product is calculated by the following method
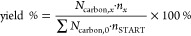
2

In this formula, *N*_carbon,*x*_ means the number of C atoms in the molecule of the product (*x*) and *n*_*x*_ is the amount of the corresponding product after the reaction. Meanwhile, *N*_carbon,0_ is the number of C atoms in the molecule of the individual reactant involved in the formation of this product (*x*), and *n*_START_ is its corresponding initial amount.

### 2.4 Characterization of Catalysts

#### 2.4.1 Aberration-Corrected Scanning Transmission Electron Microscopy

Aberration-corrected scanning transmission electron microscopy (AC-STEM) was performed using a Thermo Fisher Themis Z S/TEM, operating at 300 keV. The instrument was equipped with a high-angle annular dark field (HAADF) for high-spatial-resolution STEM–HAADF.

#### 2.4.2 Scanning Electron Microscopy

Scanning electron microscopy (SEM) was performed using a Tescan MAIA3 FEG-SEM operating at 15 kV. Samples were mounted by dry dispersion onto carbon Leit discs and analyzed uncoated. Energy-dispersive X-ray (EDX) analysis was performed using an Oxford Instruments XMAX^80^ detector and interpreted using Oxford Aztec software.

#### 2.4.3 CO-Diffuse Reflectance Infrared Fourier Transform Spectroscopy

CO-diffuse reflectance infrared Fourier transform spectroscopy (DRIFTS) data were collected on a Bruker Tensor 27 FT-IR spectrometer (spectral resolution: 4 cm^–1^), equipped with an MCT/A detector and a Praying Mantis high-temperature in situ cell (HVC-DRP-4). The MCT/A detector was cooled with liquid nitrogen before the experiment, and the number of scans for each individual spectrum was set to 64. After being swept under a flow of N_2_ (20 mL·min^–1^) for 30 min, a background spectrum was collected before a flow of 1% CO/N_2_ (20 mL·min^–1^) was introduced in the sample cell for a further 30 min. The gas flow was then changed back to N_2_ (20 mL·min^–1^), which was passed over the sample for an additional 30 min to ensure that any physisorbed CO molecules were removed. The final spectrum was recorded at the end of this desorption step, through comparison with the background spectrum taken previously.

#### 2.4.4 X-ray Photoelectron Spectroscopy

X-ray photoelectron spectroscopy (XPS) measurements were carried out on a Thermo Scientific K-Alpha^+^-photoelectron spectrometer using a microfocused monochromatic Al Kα radiation operating at 72 W (6 mA × 12 kV). Samples were pressed into the wells of a powder holder supplied by the instrument’s manufacturer. Samples were analyzed using a dual electron–ion flood gun, which gives a C–C bond-binding energy for PET at 284.8 eV, and calibration to the C (1s) peak of adventitious carbon was found to give a variance of *ca.* 0.7 eV in binding energies of the support peaks and assumed to be a consequence of the treatments involved.. Data analysis was performed using CasaXPS^28^ with Scofield sensitivity factors and an electron escape depth kinetic energy correction of 0.6, after the correction for analyzer transmission.

#### 2.4.5 X-ray Absorption Spectroscopy

X-ray absorption spectroscopy (XAS) measurements for the Pt L_3_ edge were conducted at the Canadian Light Source on the IDEAS beamline. A Pt foil was used for energy calibration. The samples were reduced in the furnace at 300 °C for 2 h under 5% H_2_/Ar (100 mL·min^–1^) and covered by Kapton film immediately after being removed from the reductive atmosphere. The conditions used for this reductive treatment were the same as those reported in Section 2.2. X-ray absorption near-edge structure (XANES) and extended X-ray absorption fine structure (EXAFS) data were analyzed using Athena/Artemis software.

#### 2.4.6 Surface Area Analysis

Surface area analysis was conducted on a Quantachrome Quadrasorb using a five-point BET method between *p*/*p*_0_ values of 0.05 and 0.3. All measurements were carried out using N_2_ at 77 K. Samples (150 mg) were degassed under vacuum for 4 h at 200 °C before analysis.

#### 2.4.7 CO-Pulse Chemisorption

CO-pulse chemisorption was performed on a Micromeritics AutoChem II 2920 instrument equipped with a thermal conductivity detector (TCD). The method used was based on that of Tanabe et al.^29^ A sample was added to a U-shaped quartz tube between two pieces of quartz wool and heated to 200 °C (ramp rate: 5 °C·min^–1^) in a flow of 10% H_2_/Ar (50 mL·min^–1^). These conditions were maintained for 1 h. After 1 h, the temperature was maintained, but the carrier gas was changed to argon (50 mL·min^–1^) for a further hour to purge the surface of hydrogen. The sample was subsequently cooled to room temperature and then to 195 K in a dry ice–ethanol bath, at which point the carrier gas was changed to helium (50 mL·min^–1^). The TCD baseline was allowed to stabilize before pulses of 1% CO/He were injected until the peak area remained constant. During the analysis, dry ice was added to the slurry periodically to ensure that the temperature was equal to 195 ± 3 K.

#### 2.4.8 CO_2_/NH_3_ Temperature-Programed Desorption Measurements

CO_2_/NH_3_ temperature-programed desorption (CO_2_-/NH_3_-TPD) measurements were implemented on a CHEMBET TPR/TPD chemisorption analyzer (Quantachrome Industries) with TCD. During the experiments, the CeO_2_ support was first heated in a He flow (100 mL·min^–1^) at 300 °C (ramp rate: 10 °C·min^–1^) for 1 h on the facility, while the 1% Pt/CeO_2_ catalysts were in situ reduced under 10% H_2_/Ar (100 mL·min^–1^) at 300 °C for 1 h and then swept in a He flow (100 mL·min^–1^) for an additional hour at the same temperature. The samples were exposed to CO_2_ at 60 °C or to NH_3_ at 90 °C for 30 min adsorption. After the saturation of the adsorbent over the surface of the sample, the sample was swept by He (100 mL·min^–1^) for 1 h to eliminate the physisorption of the probe molecules. The TPD profiles were recorded at the ramp rate of 10 °C·min^–1^ in a 100 mL·min^–1^ He gas flow.

#### 2.4.9 Inelastic Neutron Scattering Study

Inelastic neutron scattering (INS) study was carried out on the TOSCA at the ISIS Facility, STFC Rutherford Appleton Laboratory, UK. All the INS spectra in this study were recorded when the sample was cooled and stabilized at a temperature below 30 K.

In this study, a 2 wt % Pt/CeO_2_ catalyst (20 g) was loaded into a flow-type stainless-steel cell, which can be used as a static cell with all valves closed. (NH_3_)_4_Pt(NO_3_)_2_ was used as the Pt precursor for the preparation of the 2% Pt/CeO_2_ catalyst in this experiment. The catalyst was first heated at 300 °C (ramp rate: 5 °C·min^–1^) under He (150 mL·min^–1^) for 3 h to remove the remaining trace water. Following this, the activated catalyst was reduced under a H_2_ flow at 300 °C for 2 h. After that, the reduced sample was swept under He (150 mL·min^–1^) for 15 min at 300 to remove the adsorbed H species. The sample was cooled to <30 K using a closed-cycle refrigerator cryostat during data collection. The dehydrogenation of 2-propanol in the inert atmosphere was chosen as the probe reaction to investigate the mechanism of alcohol dehydrogenation. 2-Propanol was bubbled into the cell with He (150 mL·min^–1^) for 1 h at room temperature to saturate the surface of the catalyst. Following this, the cell was sealed and cooled down to collect the spectrum of the initial state of the catalytic system. The system was then heated to 180 °C and held for 2 h. After that, the cell was again cooled to 10 K to collect the spectrum of the final state over the catalyst. Finally, the cell was warmed to room temperature and flushed with He (150 mL·min^–1^); the exhaust gas was analyzed using a mass spectrometer.

#### 2.4.10 DFT Calculations

DFT calculations were carried out using the Vienna ab initio simulation package^30,31^ with the PBE functional under the generalized gradient approximation.^32^ We applied a Hubbard *U* correction with an effective *U* value of 5.0 eV on the Ce 4f orbitals to describe the localized electronic states accurately.^33,34^ The projector-augmented wave^35,36^ method was used to represent the core–valence interactions; the H (1s), C(2s, 2p), O (2s, 2p), Ce (4f, 5s, 5p, 5d, 6s), and Pt (4f, 5s, 5p, 5d, 6s) shells were treated as valence electrons. The kinetic energy cutoff was set to 400 eV. For the calculation on the unit cell of ceria, a 4 × 4 × 4 *k*-point mesh was used for the Brillouin zone integrations. The calculated lattice parameter of ceria (5.450 Å) is in good agreement with the corresponding experimental value (5.411 Å).^37^

Recent studies reported that surface reconstruction occurs on the polar CeO_2_(100) surface and both O-termination and CeO_4_-termination reconstructions are present on the surface.^38−40^ Since Pt prefers bonding to the surface O, the widely used O-terminated reconstructed CeO_2_(100) surface should be sufficient for modeling the Pt–CeO_2_(100) interface and thus is used for the present study. The O-terminated reconstructed CeO_2_(100) surface slab contains seven atomic layers and a large vacuum gap (>10 Å) to avoid interactions between slabs. For CeO_2_(100)-supported Pt raft/nanoparticle species, the surface was extended at a (5 × 5)/(4 × 4) cell. For surface calculations, a 1 × 1 × 1 *k*-point mesh was used for the Brillouin zone integrations. The bottom three atomic layers were fixed during geometry optimizations to estimate bulk properties. A constrained optimization scheme^41,42^ was used to search the transition state (TS) structures. Geometry optimizations were finished until the Hellman–Feynman force on each relaxed ion was less than 0.05 eV/Å.

The adsorption energies (*E*_ads_) of CPL were calculated as follows
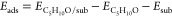
where *E*_sub_, , and  are the DFT total energies of the substrate, a gas-phase C_5_H_10_O molecule, and the adsorption complex, respectively.

## 3 Results and Discussion

### 3.1 Catalyst Synthesis and Testing

**Figure 1:**
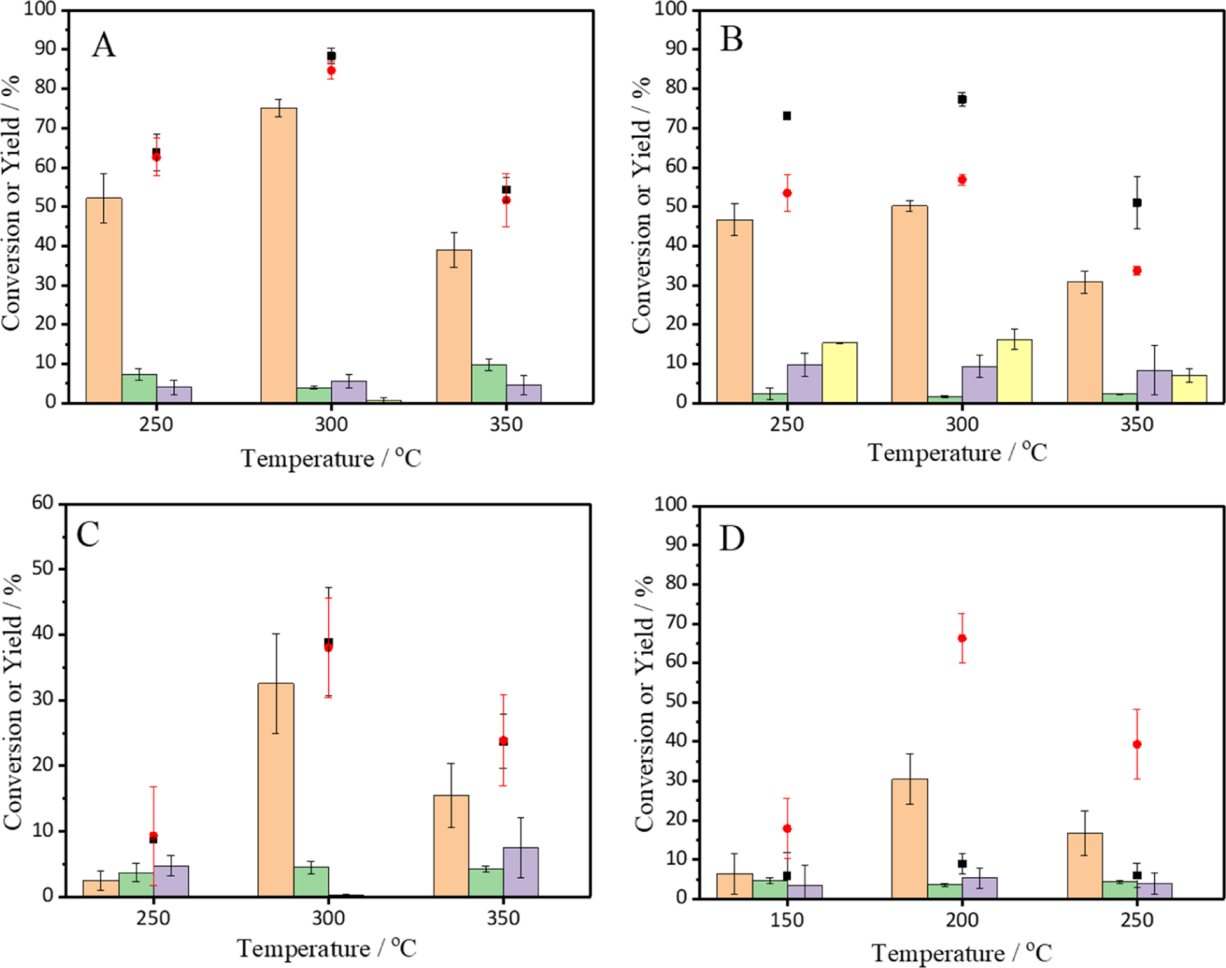
Optimization of the reduction temperature of 1% Pt/CeO_2_ catalysts: (A) 1% Pt/CeO_2_-DE-H; (B) 1% Pt/CeO_2_-DE-N; (C)1% Pt/CeO_2_-AA-H; and (D)1% Pt/CeO_2_-Sigma-H. Conditions: 2 mmol CPL, 2 mmol CPA, 50 mg of catalysts, 300 μL of decane (Int.S), 10 mL of *p*-xylene (solvent), 140 °C, and 800 rpm; reaction time: (A) 2; (B) 2; (C) 3; and (D) 16 h. Key: CPL (black square); CPA (red diamond); dicyclopentylamine (orange bar); *N-Cyclopentylcyclopentanamine* (green bar); cyclopentanone (purple bar); and others (yellow bar).

To begin, a series of Pt/CeO_2_ catalysts were synthesized through the impregnation of three CeO_2_ supports (AA, Sigma, and DE) with either H_2_PtCl_6_ (−H) or (NH_3_)_4_Pt(NO_3_)_2_ (−N). Each of the impregnated materials was subsequently thermally reduced at temperatures between 150 and 350 °C under a flow of 5% H_2_/Ar. Their performance as catalysts in a cascade reaction involving alcohol dehydrogenation, amine and ketone coupling, and hydrogenation (Scheme 1) was then assessed. The results from these experiments are illustrated in Figure 1 and listed in Table 2. Note that these reactions were run for different lengths of time owing to differences in the activity exhibited by the catalysts.

**Table 2:** Catalytic Performance of the CeO_2_-DE Support, Unreduced 1% Pt/CeO_2_, and 1% Pt/CeO_2_ Catalysts Reduced at Optimized Temperature^a^

		Conv. (%)		Yield %			
Catalyst	Time (h)	CPL	CPA	DCPA	DCPI	CPO	Others
1% Pt/CeO_2_-DE-H-300	2	88.4 ± 2.0	84.7 ± 2.2	75.0 ± 2.2	3.8 ± 0.4	5.5 ± 1.8	0.6 ± 0.6
1% Pt/CeO_2_-DE-N-300	2	77.3 ± 1.8	56.9 ± 1.4	50.1 ± 1.4	1.5 ± 0.3	9.2 ± 2.8	16.1 ± 2.7
1% Pt/CeO_2_-AA-H-300	3	38.9 ± 8.3	38.0 ± 7.6	32.5 ± 7.6	4.4 ± 0.9	0.2 ± 0.2	0.0
1% Pt/CeO_2_-Sigma-H-200	16	9.0 ± 2.5	67.3 ± 6.3	30.4 ± 3.4	3.5 ± 0.4	5.2 ± 2.6	0.0
1% Pt/CeO_2_-DE-H-unreduced	3	0.0	0.0				
CeO_2_-DE	3	0.0	0.0				

a Conditions: 2 mmol CPL, 2 mmol CPA, 81.3 mmol *p*-xylene (solvent), 140 °C, 50 mg of catalysts, 1.54 mmol decane (Int.S), 800 rpm, and 2 bar N_2_. Key: CPL (cyclopentanol); CPA (cyclopentylamine); DCPA (dicyclopentylamine); DCPI (*N*-cyclopentyl-cyclopentanimine); CPO (cyclopentanone); and others {[1,1′-bi(cyclopentan)]-2-one and [1,1′-bi(cyclopentan)]-2-ol}.

The rate of CPL dehydrogenation (per gram of the catalyst) was notably higher over Pt impregnated onto the CeO_2_-DE supports. This appeared to be the case irrespective of the Pt salt used, although slightly higher levels of CPL and CPA conversion were observed over the Pt/CeO_2_-DE-H catalyst. The temperature used for the reductive heat treatment also had a notable impact on substrate conversion. Higher CPL conversion over the Pt/CeO_2_-DE-H, Pt/CeO_2_-DE-N, and Pt/CeO_2_-AA-H catalysts was observed when these materials were reduced at 300 °C. For the Pt/CeO_2_-Sigma-H catalyst, the optimum reduction temperature was significantly lower (200 °C). Another interesting observation is that the comparative rates for the consumption of the two substrates (CPL and CPA) differ depending on the catalyst used. For the Pt/CeO_2_-DE-H and Pt/CeO_2_-AA-H catalysts, the rate of CPL consumption was only slightly lower than the rate of CPA consumption. However, a large difference in the rates of CPA and CPL conversion was observed over the 1% Pt/CeO_2_-DE-N-300 catalyst. Control experiments conducted over the bare CeO_2_-DE support and impregnated Pt/CeO_2_-DE-H (prior to reduction) evidenced that Pt species are required for alcohol dehydrogenation, the first step in the reaction cascade.

Although the Pt/CeO_2_-DE-H and Pt/CeO_2_-DE-N catalysts exhibited comparable CPL conversions, the catalyst prepared with the chloride precursor (Pt/CeO_2_-DE-H) was notably more selective to the desired coupled product (DCPA). Instead, over the Pt/CeO_2_-DE-N catalyst, a large proportion of other products were formed, which, through utilizing GC–MS, were determined to predominantly arise from the aldol condensation of CPO and have been noted previously.^43^ It is, therefore, logical to assume that the Pt/CeO_2_-DE-N catalysts possess different physicochemical properties, compared to the Pt/CeO_2_-DE-H catalysts, which favor this transformation. Given that this side reaction may compete with the coupling of CPO and CPA, it may explain why there is also a notable gap between the rates of CPA and CPO consumption over the Pt/CeO_2_-DE-N series; CPO formed from CPL dehydrogenation is also partaking in aldol condensation reaction(s) rather than coupling with CPA. This trend in selectivity is further reflected in associated time online studies (Figure S1). Comparatively, the series of 1% Pt/CeO_2_-AA-H and the 1% Pt/CeO_2_-Sigma-H catalysts were less active than catalysts prepared using the CeO_2_ support produced through thermal decomposition of cerium (III) nitrate hexahydrate (CeO_2_-DE).

### 3.2 Identifying the Catalytically Active Species

**Figure 2:**
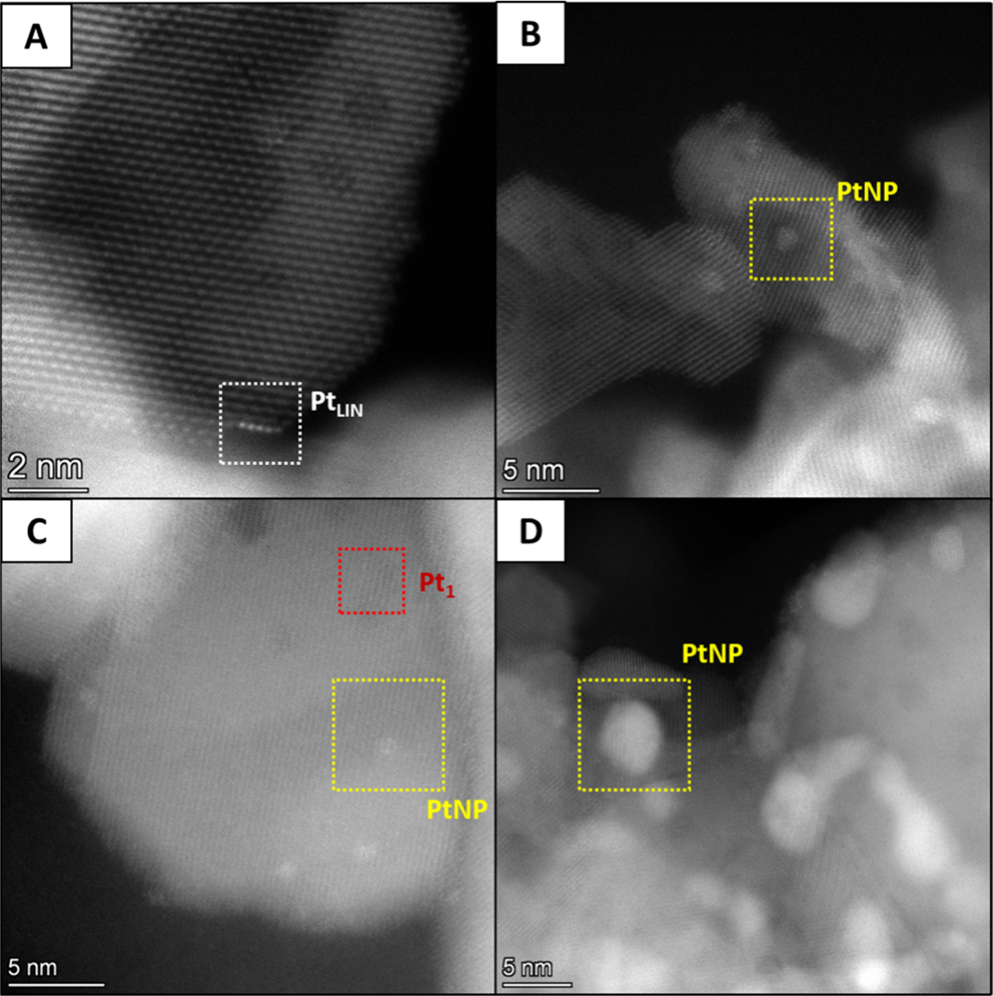
AC-STEM images of (A) 1% Pt/CeO_2_-DE-H-300; (B) 1% Pt/CeO_2_-DE-N-300; (C) 1% Pt/CeO_2_-AA-H-300; and (D)1%/CeO_2_-Sigma-H-200. Different Pt structures are clearly labeled: linear Pt species (white squares); Pt nanoparticles (yellow squares), and Pt single atoms (red squares).

Given that the series of Pt catalysts performed so differently in the reaction, it was important to characterize the materials to understand which properties influence the catalysis. Given that it would not be possible to fully characterize all 12 of the catalysts presented in Figure 1, the majority of the subsequent characterization was only conducted on the most active catalysts from each series (1% Pt/CeO_2_-DE-H-300, 1% Pt/CeO_2_-DE-N-300, 1% Pt/CeO_2_-AA-H-300, and 1% Pt/CeO_2_-Sigma-H-200). First, these catalysts were examined by AC-STEM (Figures 2, S2,and S3). Several different Pt structures were observed across the samples, which included Pt single sites (Pt_1_), Pt nanoparticles, and linear Pt species. From the microscopy acquired, it was not possible to deduce explicitly whether the latter species existed as 2D Pt rafts or singular lines of Pt atoms. The formation of both these features is conceivable; 2D Pt rafts could readily form through epitaxial deposition onto CeO_2_ (Figure S2). A recent study by Xiong et al. demonstrated that 2D Pt and Pd rafts could be synthesized through atom trapping on CeO_2_ supports.^44^ Here, the authors demonstrated that strongly bound Pt_1_ sites serve as nucleation sites and promote 2D linear growth along CeO_2_ planes. It is, however, also possible that 2D lines of Pt atoms could form. Although thermodynamically unstable on a flat CeO_2_ surface, their presence could well be stabilized through interaction with step edges on the CeO_2_ support.

Interestingly, however, the presence (and proportion) of the Pt structures differed depending on the support and Pt salt used during their synthesis. The linear Pt species (Figure 2A) were observed in the 1% Pt/CeO_2_-DE-H catalyst. Importantly, there was no evidence of these species in the analogous catalyst prepared using the nitrate precursor (Figure 2B) or on the catalysts prepared from the commercial CeO_2_ supports (Figure 2C,D), suggesting that their formation is related to the Pt precursor and support used for their preparation. However, this hypothesis does not explain why these features were not observed in the 1% Pt/CeO_2_-DE-N-300 sample and requires further consideration. Pt_1_ species were observed in the 1% Pt/CeO_2_-AA-H-300 catalyst (Figure 2C). Importantly, Pt nanoparticles were observed in all of the catalysts (Figures 2 and S3) and extremely large particles of Pt (>100 nm in diameter) were observed in low-magnification micrographs of the 1% Pt/CeO_2_-DE-H-300 and 1% Pt/CeO_2_-DE-N catalysts (Figure S2B,D, respectively).

**Figure 3:**
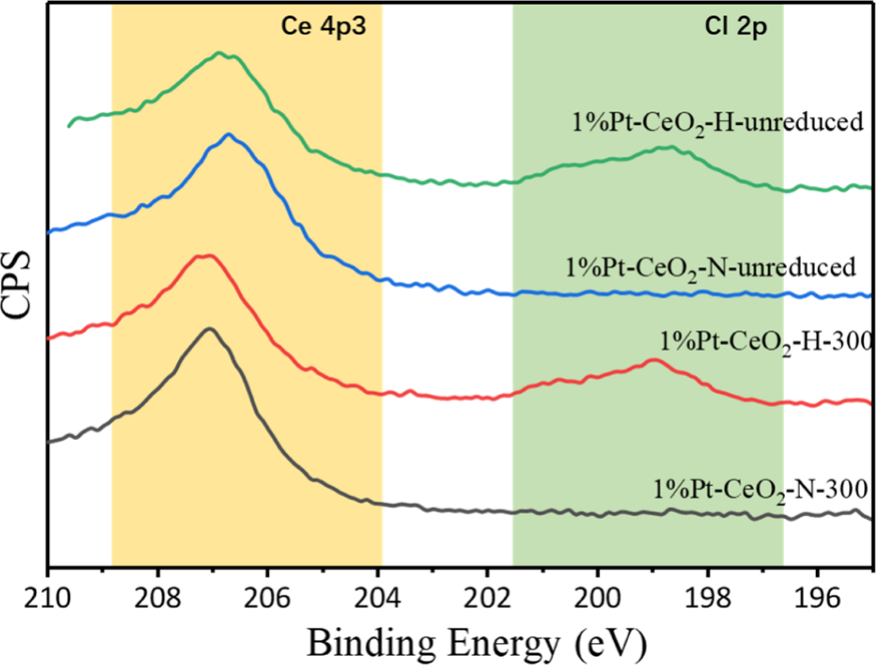
XPS spectra of Cl 2p orbit of 1% Pt/CeO_2_-DE-H and 1% Pt/CeO_2_-DE-N samples.

The variety and differing distributions of Pt species in the catalysts highlight how the properties of the CeO_2_ support and Pt precursors can significantly affect the dispersion and morphology of Pt in the final catalyst. To further understand the relationship between Pt dispersion and catalyst performance, the catalysts were examined by CO chemisorption (Table S1). TOFs of 3751, 2736, 242, and 13 h^–1^ were determined for the DE-H, DE-N, AA, and Sigma-supported catalysts, respectively. Given that the Pt/CeO_2_-DE-H catalyst possessed both linear Pt species and Pt NPs, the analogous Pt/CeO_2_-DE-N catalyst only possessed Pt NPs, which could indicate that the former species are more effective at promoting CPA dehydrogenation. Interestingly, the catalyst that was found to possess a large proportion of Pt_1_ species exhibited exceptionally low TOFs, suggesting that these species are not effective at promoting CPA dehydrogenation. Given that the 1% Pt/CeO_2_-Sigma-H-200 and the 1% Pt/CeO_2_-AA-H-300 catalysts exhibited such poor performance, the remaining characterization conducted focused specifically on the 1% Pt/CeO_2_-DE-H-300 and 1% Pt/CeO_2_-DE-N-300 catalysts. A key factor was understanding the properties that led to differences in the reaction selectivity (Figures 1 and S1 and Table 2). To establish whether this was a function of Cl in the catalysts, both catalysts (before and after reduction) were probed by XPS. The Cl 2p region (Figure 3) clearly evidences that Cl^–^ (BE = 199 eV) is present in the 1% Pt/CeO_2_-DE-H-300 catalyst before and after reduction. This was supported by additional EDX analysis (Table S2). Quantification of the elemental composition confirmed that a significant proportion of Cl^–^ was present in the reduced catalyst (atomic ratio of Cl/Pt = 6). Given that the precursor used for the synthesis of this catalyst was H_2_PtCl_6_, it is suggested that the reductive heat treatment had no effect on the removal of Cl^–^ from the catalyst. Previously, researchers have observed that cerium oxychloride can be formed through the thermal reduction of noble metal chloride precursors supported on lanthanide oxide supports.^45−47^ Although some of the chlorides are likely to be retained in the form of cationic Pt, it is possible that some will also be present as cerium oxychloride.

**Figure 4:**
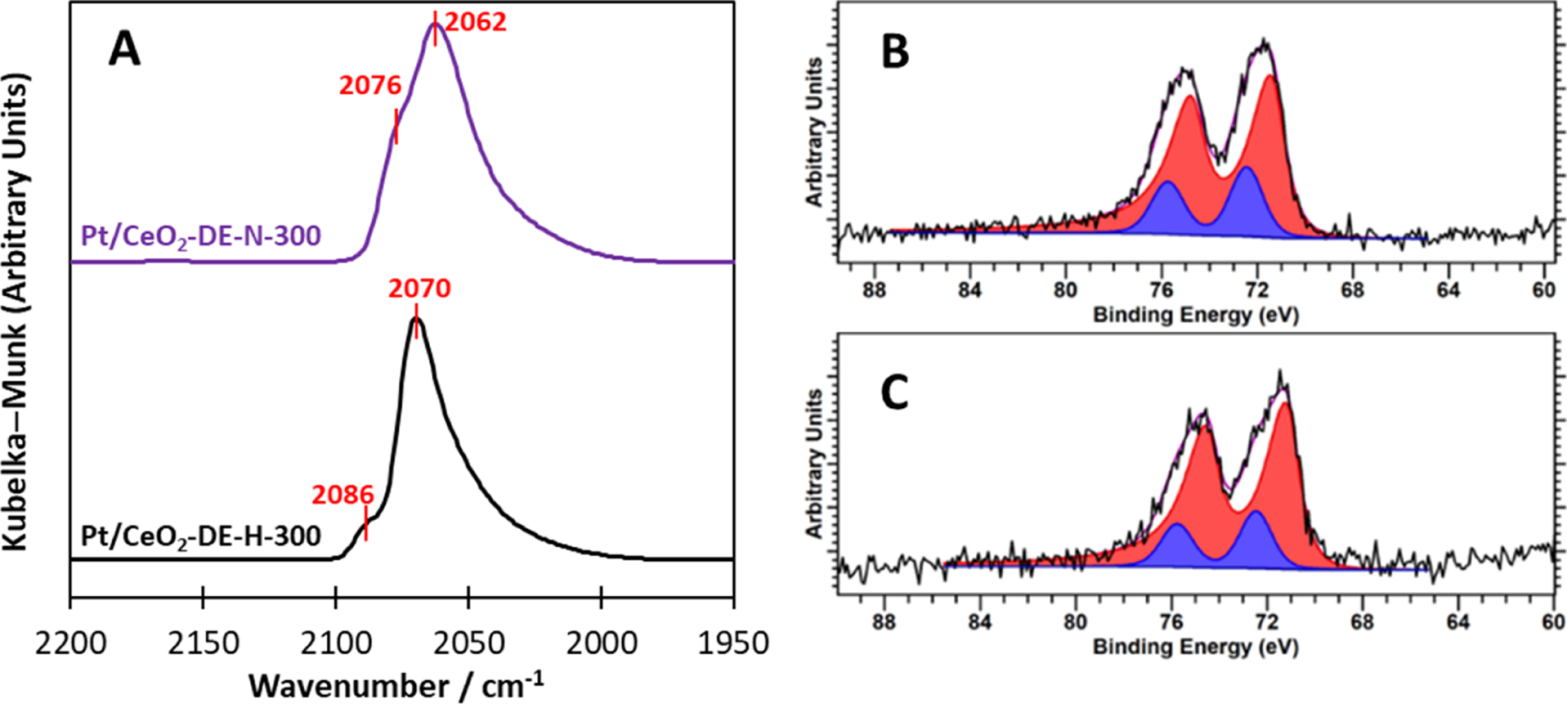
(A) CO-adsorption DRIFTS spectra for the Pt/CeO_2_-DE-H-300 and Pt/CeO_2_-DE-N-300 catalysts. (B and C) 4f core-level photoelectron spectra of the Pt/CeO_2_-DE-H-300 and Pt/CeO_2_-DE-N-300 catalysts, respectively, where red = Pt^0^ and blue = Pt^2+^.

Next, the chemical state of Pt in these catalysts was probed as it is widely acknowledged that this can dramatically influence the activity and/or selectivity of supported metal catalysts.^48,49^ First, both catalysts were subjected to CO-DRIFTS experiments; the corresponding spectra are presented in Figure 4. Two notable adsorption bands were observed in both spectra, although their energies and relative proportions were different. In the spectrum of the 1 % Pt/CeO_2_-DE-H-300 catalyst, the two vibrational peaks were centered at 2088 and 2074 cm^–1^, and the lower energy vibration was significantly larger. On the contrary, in the CO-DRIFTS spectrum of the 1 % Pt/CeO_2_-DE-N-300 catalyst, both vibrational peaks exhibit lower energy (centered at 2076 and 2062 cm^–1^). Considering the reference literature, we attribute the higher energy vibrations (2088 and 2076 cm^–1^) to linear CO adsorbed onto Pt species possessing high coordination numbers and the lower energy vibration (2074 and 2062 cm^–1^) to linear CO adsorption onto Pt sites with lower coordination numbers, such as kinks and steps.^50−53^ Importantly, no vibrations indicative of CO adsorption onto cationic Pt_1_ species were observed in both spectra. This is in stark contrast to the Pt/CeO_2_-Sigma-H-200 and Pt/CeO_2_-AA-H-300 catalysts (Figure S4), which exhibited large peaks centered at 2117 and 2115 cm^–1^, respectively. Pt–CO stretches at these energies are characteristic of CO adsorption on cationic Pt,^54,55^ which supports the observation of single Pt atoms in the analogous STEM analysis of these materials (Figure 2C,D). This suggests that Pt_1_ species are not common in these catalysts. However, the large proportion of low-coordinate Pt observed in the 1% Pt/CeO_2_-DE-H-300 catalyst could be attributed to the linear Pt species it possesses (Figures 2A and S2B), which were not observed in the analogous 1 % Pt/CeO_2_-DE-N-300 catalyst (Figure 2B). It is also important to consider why both the linear CO–Pt bands exhibited in the spectrum of the 1% Pt/CeO_2_-H-300 catalyst exhibit higher energy. Such behavior is often indicative of an electronic effect, which can be induced through metal polarization.^56^ Given that there is an abundance of residual Cl^–^ present on the surface of the 1% Pt/CeO_2_-DE-H-300 catalyst (Figure 3 and Table S2), it is possible that this might be the origin of this effect.

To investigate this further, the Pt 4f core-level photoelectron spectra of the two catalysts were assessed (Figure 4C,D). Evidently, there is a mixture of oxidation states in both materials, however, we do not discount the presence of small quantities of Pt (II). For these catalysts, a Pt 4f_7/2_ state is found at 71.4 eV (±0.2 eV) characteristic of metallic Pt, with a second species at *ca.* 72.5 eV, typically characteristic of Pt(II). Although we observed two states of chlorine (Cl 2p_3/2_ 197.8 and 198.7 eV) on the catalysts prepared with a chloride precursor (Figure S5), we did not assign the Pt(II) species as a discrete PtCl_*x*_ species since the binding energy Pt state is lower than that of Pt in K_2_PtCl_6_ (75.3 eV). Although similar to that of K_2_PtCl_4_ (72.8 eV) as measured on the same system, the corresponding metal–chlorine signal (198.7 eV) is far in excess of the Pt(II) component, with ratios showing a 20- to 30-fold excess and the chlorine likely associated with the ceria support. Therefore, we believe that this Pt (II) state is likely to be indicative of a partially reduced Pt oxide species or (OH)_2_. From inspection of the Ce 3d region, it was not possible to deduce whether different proportions of Ce^3+^ species were present in the different catalysts due to sample reduction during the analysis.

**Figure 5:**
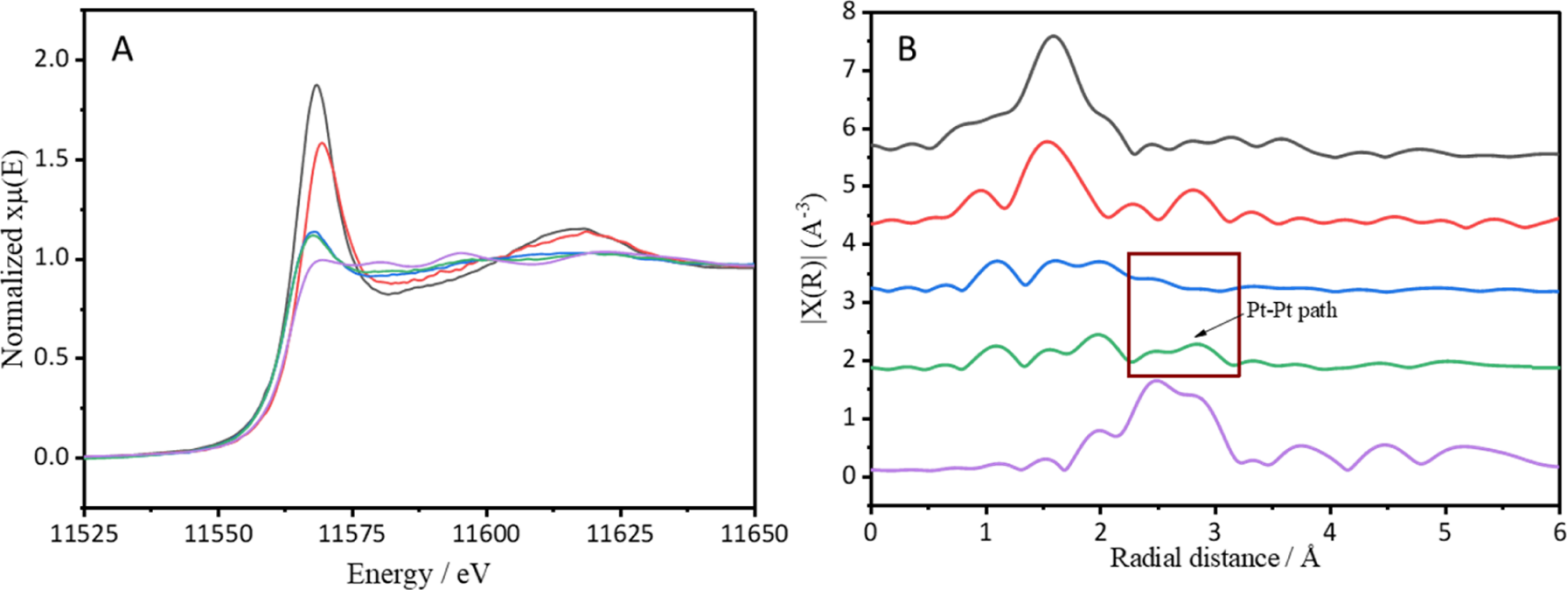
(A) XANES spectra; (B) EXAFS spectra in the *R* space of reduced and unreduced 1% Pt/CeO_2_-DE-H and 1% Pt/CeO_2_-DE-N samples (Pt foil used for energy calibration). Key: unreduced 1% Pt/CeO_2_-DE-H (black line); unreduced 1% Pt/CeO_2_-DE-N (red line); 1% Pt/CeO_2_-DE-H-300 (blue line); 1% Pt/CeO_2_-DE-N-300 (green line); and Pt foil (purple line).

To complement the XPS studies, the influence of the metal precursor on the electronic states of Pt species was studied by XAS for the 1% Pt/CeO_2_-DE-H-300 and 1% Pt/CeO_2_-DE-N-300 catalysts. For comparative purposes, XAS experiments were also conducted on a Pt foil and the corresponding unreduced catalysts. The Pt L_3_-edge XANES spectra of each sample are presented in Figure 5A. The height of the white line in these samples significantly decreases after the reductive pretreatment, further evidencing that the majority of Pt present is in a reduced state.^49^ The white line in the spectrum of the Pt/CeO_2_-DE-H-300 catalyst is, however, slightly higher than that observed in the spectrum of the 1% Pt/CeO_2_-N-300 sample. Through consideration of the corresponding CO-DRIFTS and XPS data (Figure 4), this is likely attributed to the different electronic properties of the Pt species in the two catalysts.

The corresponding EXAFS spectra of these samples, in the R space, are presented in Figure 5B. Once again, these spectra suggest that the Pt present in both the final catalysts is reduced. A notable signal in the range of 2∼3 Å is also observed in the spectrum of the Pt/CeO_2_-DE-N-300 catalyst. No such signal is observable in the corresponding reduced catalyst prepared using the H_2_PtCl_6_ precursor. This suggests that Pt species present in the Pt/CeO_2_-DE-N-300 catalyst possess a more typical metallic Pt character. The absence of this characteristic Pt–Pt signal in the EXAFS spectrum of 1% Pt/CeO_2_-DE-H-300 catalysts also supports the conclusions obtained from the microscopy and CO-DRIFTS experiments: the 1% Pt/CeO_2_-DE-H-300 catalyst possesses a greater proportion of low-coordinate Pt species.

### 3.3 Derivation of the Surface Mechanism

**Figure 6:**
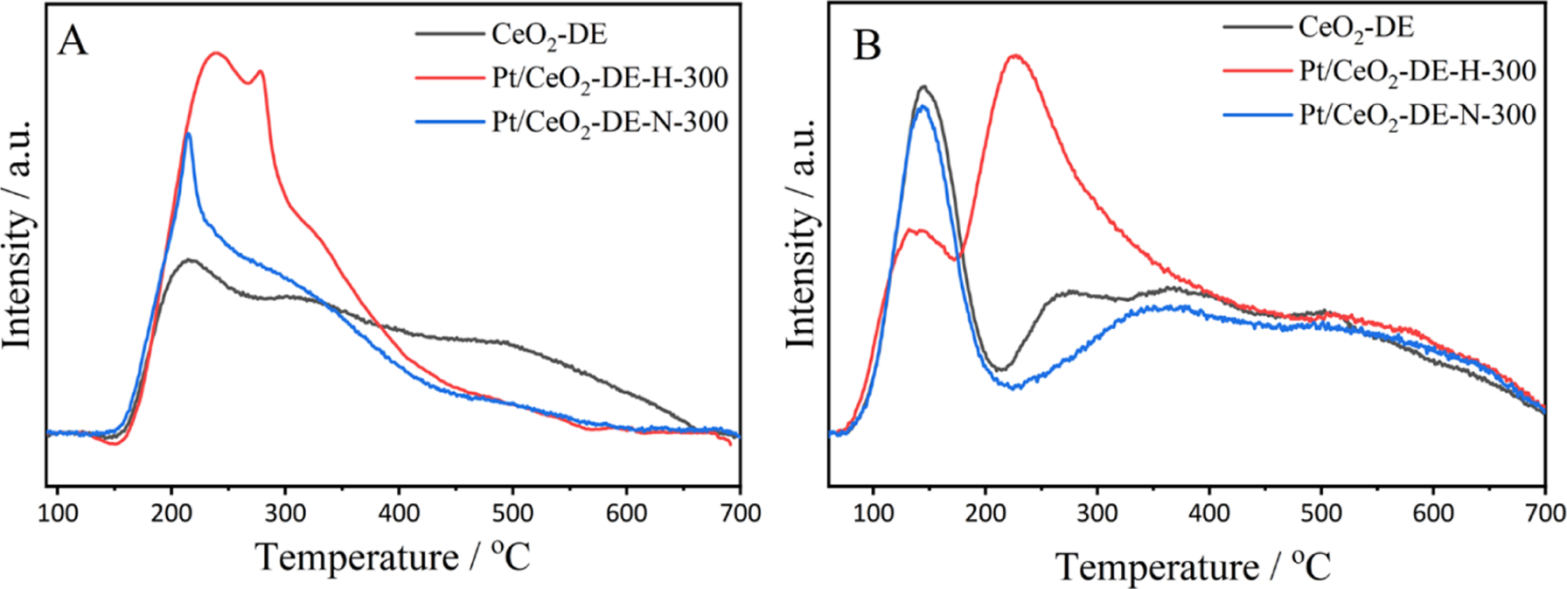
(A) NH_3_-TPD and (B) CO_2_-TPD profiles of pristine CeO_2_-DE, 1% Pt/CeO_2_-DE-N-300, and 1% Pt/CeO_2_-DE-H-300 catalysts.

It is well-established that acid and base sites in heterogeneous catalysts can play an important role in directing surface mechanisms. Given that the 1% Pt/CeO_2_-DE-H-300 and 1% Pt/CeO_2_-DE-N-300 exhibited different selectivities (Figure 1 and Table 2) in this reaction, it was important to consider how the residual Cl^–^ present might influence this. To assess whether the residual Cl^–^ influenced the acid and basic properties, a series of NH_3_- and CO_2_-TPD experiments were conducted on the 1% Pt/CeO_2_-DE-H-300 and 1% Pt/CeO_2_-DE-N-300 catalysts, the profiles of which are displayed in Figure 6. Analogous experiments conducted on the pristine CeO_2_-DE support were also performed for comparison. The NH_3_-TPD profiles (Figure 6A) indicate that after impregnation and reduction of Pt, there is an increase in the proportion of weak and medium acid sites (*T* < 400 °C) and a decrease in the quantity of strong acidic sites (*T* > 400 °C); this is most obvious for the Pt/CeO_2_-DE-H-300 catalyst, prepared using the H_2_PtCl_6_ precursor. More revealing information was, however, acquired when the basicity of the samples was probed using CO_2_ TPD (Figure 6B). Compared to the pristine CeO_2_-DE support, only a small reduction in the population of basic sites is observed in the profile of the Pt/CeO_2_-DE-N-300 catalyst. However, some more notable differences are observed in the profile of the Pt/CeO_2_-DE-H-300 catalyst: a peak centered at *ca.* 150 °C, which is characteristic of weak basic sites, decreases and a dramatic increase is observed in a peak centered at *ca.* 250 °C, which is characteristic of medium-strength basic sites.^57^ Tentatively, we attribute these medium-strength base sites to the presence of the CeOCl phase on the surface of these materials. The use of chlorinated reagents in the preparation of CeO_2_-supported catalysts has previously been linked with the formation of this phase,^58−60^ which is known to be stable under reductive conditions.^60^

The differences in basic properties in the two catalysts provide insights into why different product selectivity is observed in the reactions. Although it is widely accepted that aldol condensation reactions can be promoted by both acidic and basic sites, a recent study has suggested that weak Brønsted basic sites are particularly efficient at promoting such reactions.^61−64^ This is consistent with experimental testing data presented in this study. Over the Pt/CeO_2_-DE-N-300 catalyst, which possesses a significantly larger proportion of weak basic sites (Figure 6B), more condensation products are observed (Figures 1 and S1 and Table 2).

**Figure 7:**
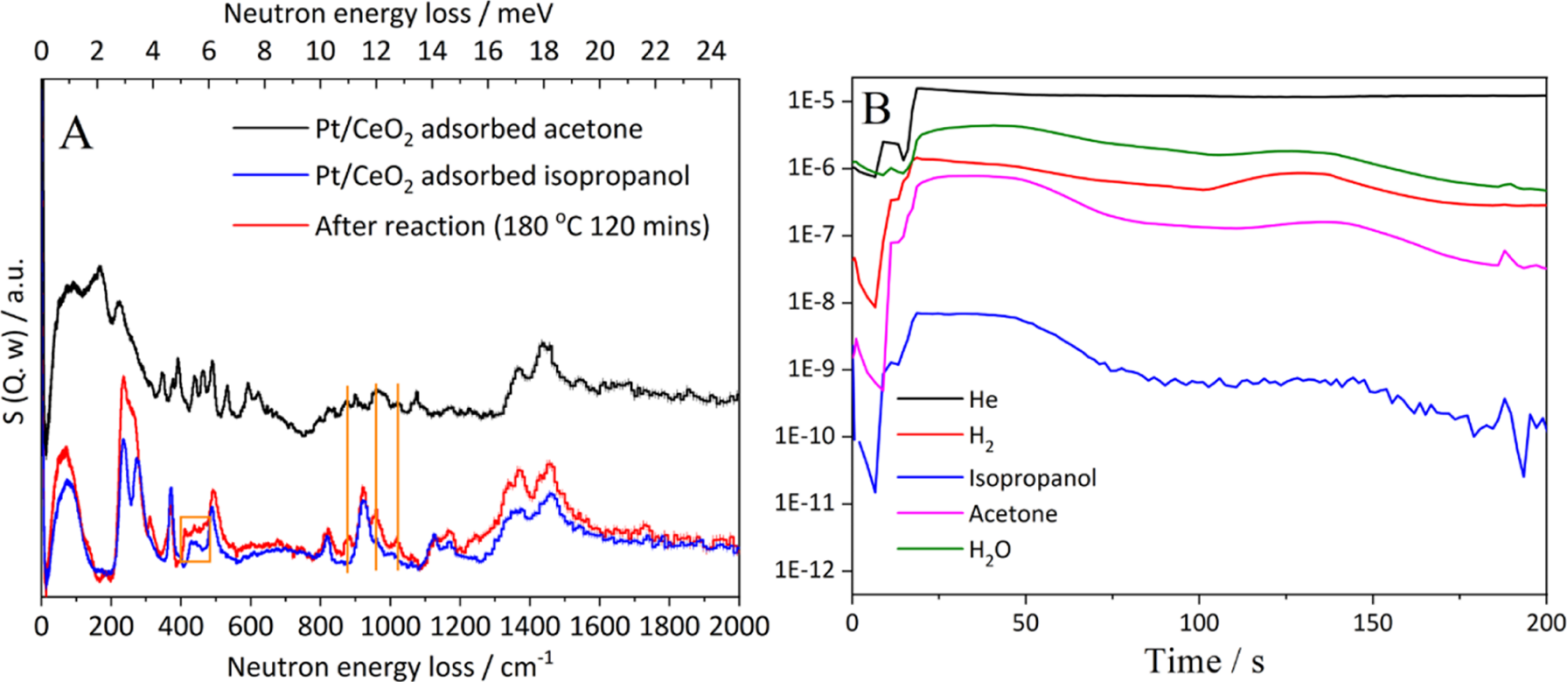
INS–MS results of the dehydrogenation of isopropanol: (A) INS spectra of acetone adsorbed on 2% Pt/CeO_2_, isopropanol adsorbed over 2% Pt/CeO_2_, and adsorbed species remaining after the reaction and (B) MS spectra of the gas-phase reaction mixture after the reaction.

The dehydrogenation of the alcohol component is considered to be the RDS in this reaction.^65,66^ A previous study involving in situ DRIFTS experiments has already illustrated that the dehydrogenation of alcohols over Pt/CeO_2_ catalysts begins with the dissociative adsorption of hydroxyl groups onto Lewis acid–base pairs, which are adjacent to Pt particles/clusters.^18^ Thus, it is proposed that the active site is likely to be situated on Pt–Ce interface sites. Here, we have conducted INS–MS measurements to further probe the mechanism (Figure 7). These experiments were conducted to evaluate intermediates formed during the dehydrogenation of isopropanol to acetone. 2-Propnanol was selected as a suitable model substrate for the dehydrogenation reaction (in place of CPL) due to its comparatively high vapor pressure. To increase the intensity of the observable signal in these experiments, an analogous 2 wt % Pt/CeO_2_ catalyst was used, which was synthesized using a Pt nitrate metal precursor. After 2 hours of the reaction at 180 °C, the INS spectra clearly show the formation of acetone. More interestingly, however, another signal at *ca.* 470 cm^–1^ is observed, which is characteristic of the Pt hydride moiety.^67^ This is likely to be formed through the abstraction of an α-H from the adsorbed alkoxy group of the isopropanol. Analogous analysis by MS during the reaction (Figure 7B) clearly shows that H_2_ gas is produced, suggesting that recombination of H and subsequent desorption of H_2_ also occur.

**Figure 8:**
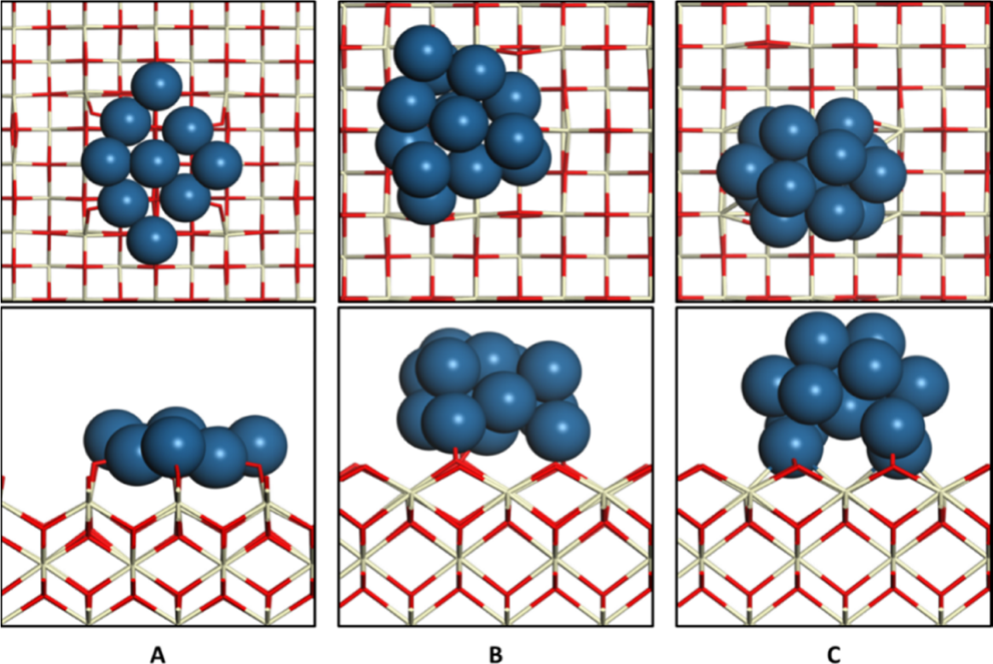
Optimized structures (top panel, top view; bottom panel, side view) of (A) model I, (B) model II, and (C) model III. Cerium, ivory; oxygen, red; and platinum, blue.

To assess how the morphology of Pt species was in the reaction, we conducted a series of theoretical calculations. Given that the high-resolution TEM (HR-TEM) experiments (Figures 2 and S2) confirmed that the majority of the Pt species resided on CeO_2_(100) surfaces, it seemed logical to use this CeO_2_ surface to construct our theoretical models. Accordingly, we constructed three different models of CeO_2_(100)-supported Pt to understand the structure–activity relationships in the Pt/CeO_2_ catalysts. In model I, the supported Pt cluster has a raft-like 2D structure and is composed of nine Pt atoms, while in models II and III, the supported Pt clusters are 13-atom nanoparticles having the reported configurations;^69−71^ as one can see, the Pt cluster in model III having a nearly spherical structure can better represent three-dimensional (3D) Pt nanoparticles than the one in model II (see Figure 8). The calculated average charge of Pt in model I is 0.194 (see Table S3), indicating that a 2D Pt raft interacts strongly with the CeO_2_ support and donates electrons to the support. In contrast, the calculated average charge of Pt in model III is −0.003 only, and only the five interfacial Pt atoms in this model have noticeable charges (see Table S3). Not surprisingly, the calculated average charge of Pt in model II (0.094) lies in between the corresponding values of models I and III (see Table S3), suggesting that the Pt cluster in model II can be regarded as the transition structure of the Pt raft in model I and the 3D Pt nanoparticle in model III.

**Figure 9:**
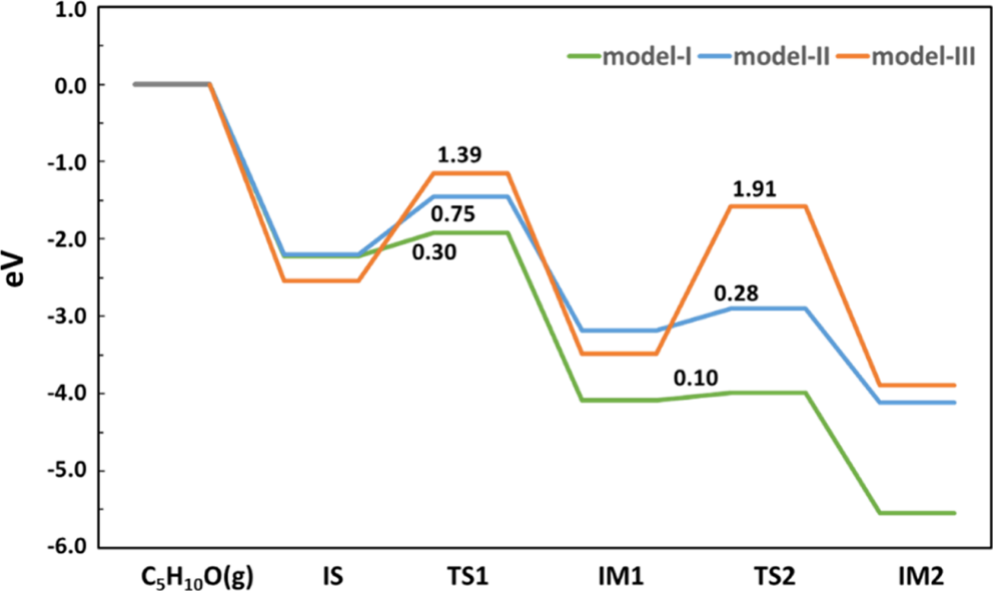
Calculated energy profiles for the dehydrogenation of CPL to CPO on models I (green), II (blue), and III (orange). C_5_H_10_O(g), IS, IM1, and IM2 represent the states of the gas-phase C_5_H_10_O molecule, adsorption of the C_5_H_10_O molecule, co-adsorption of the C_5_H_9_O species and a H atom, and co-adsorption of the C_5_H_8_O species and two H atoms, respectively; TS1 and TS2 are the corresponding TSs. The energy barriers are also given.

To further understand the structure–activity relationship, we then continued to study the key reaction, that is, the dehydrogenation of CPL (C_5_H_10_O) to CPO (C_5_H_8_O), on models I, II, and III. This reaction includes two elementary steps, namely, (i) the O–H bond breaking and (ii) the C–H bond breaking steps. The calculated energy profiles are presented in Figure 9 and the corresponding structures are given in Figures S6–S8. Among the three models, model I with a Pt raft gives the best performance for the dehydrogenation of CPL to CPO according to the calculated energetics. Although the adsorption processes of CPL on the three models are all very strong with adsorption energies ranging from −2.54 to −2.20 eV, the energy barriers for the O–H and C–H bond breaking steps on these models are quite different [model I vs model II vs model III: 0.30 vs 0.75 vs 1.39 eV (O–H bond breaking) and 0.10 vs 0.28 vs 1.91 eV (C–H bond breaking)], and model I gives the lowest barriers for both of the two elementary steps. In addition, model I also gives the most exothermic reaction energies for the two elementary steps (see Figure 9). These results suggest that the CeO_2_(100)-supported Pt raft is highly active for this reaction. Previous studies proposed that a catalyst with a stronger electron-withdrawing capability generally gives better catalytic activities toward dehydrogenation reactions.^72^ Accordingly, the higher activity of the CeO_2_(100)-supported Pt raft toward the dehydrogenation of CPL to CPO can be attributed to the unique electronic property of this catalyst that all Pt atoms are interfacial atoms having noticeable positive charges and strong electron-withdrawing capabilities (see Table S3).

**Scheme 2:**
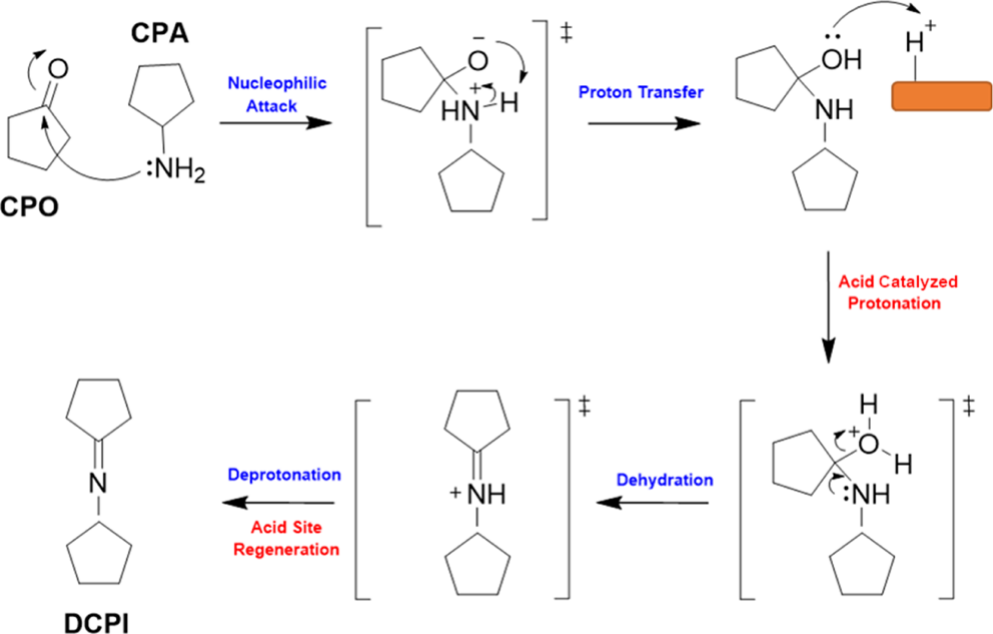
Imine formation (DCPI) Is achieved through the Coupling of CPO and CPA The important role of Brønsted acid sites on the Pt/CeO_2_ catalyst is highlighted.

In our proposed mechanism (Scheme 2), the alcohol substrate CPL undergoes dissociative adsorption on a Lewis acid–base pair on the support before an α-hydride undergoes reductive elimination onto an adjacent Pt species and leads to the formation of CPO.^68^ Given that the hydride transfer in such reactions is considered to be rate limiting, the morphology of the supported Pt species will undoubtedly have a significant impact on the rate of CPL conversion. From the testing data presented (Figures 1 and Tables 2 and S1), STEM analysis (Figures 2 and S2), in situ CO-DRIFTS experiments (Figures 4A and S3), and analogous modeling data (Figure 9), we can propose (with confidence) that the activity of the Pt species present in these materials is of the order 2D Pt > Pt nanoparticles > cationic Pt. After dehydrogenation is achieved, CPO is subjected to a nucleophilic attack from the nitrogen lone pair in the amine (CPL), leading to the formation of an unstable (polar) intermediate. Next, a proton is rapidly transferred to the electron-rich alkoxy moiety from the adjacent amine group and the former is subsequently protonated through interaction with a Brønsted acid site on the support, leading to its rapid dehydration and the formation of the corresponding Schiff base (DCPI). The DCPI can subsequently undergo reduction through interaction with Pt-adsorbed hydrogen species, leading to the formation of DCPA. Notably, a bimolecular aldol condensation reaction between two CPO molecules is in direct competition with imine formation, and it is the relative rates of these two reactions that ultimately dictate reaction selectivity. It is well-established that these reactions can be promoted by either acid or base sites and the product of this condensation can subsequently be reduced to components assigned as “other” in Scheme 1. Given that imine formation is promoted solely by Brønsted acidity, it is no surprise that the Pt/CeO_2_-DE-H catalyst is more effective at promoting imine formation as this material comprises significantly more Brønsted acid sites than the analogous Pt/CeO_2_-DE-N catalyst (Figure 6). It should be noted that small quantities of dissociated hydrogen can also recombine and desorb as H_2_ (Figure 7B), though this process is likely to be extremely limited in reactions where CPL is present due to the consumption of surface adsorbed hydrogen in subsequent reduction processes.

To assess how stable the linear Pt species were in the Pt/CeO_2_-DE-H-300 catalyst during the reaction, STEM was used to probe the catalyst after 0.5 and 2 h of the reaction (Figure S9). Linear Pt species were clearly observed in both samples, confirming that these species are, at least partially, stable under reaction conditions.

## 4 Conclusions

In this study, we assessed how the Pt metal precursor, the CeO_2_ support, and reductive conditions influenced the performance of 1 wt % Pt/CeO_2_ catalysts in hydrogen-borrowing amination reactions. The support and reduction conditions were found to have a profound effect on catalyst performance, which was attributed to the nature of the Pt components present in the final catalyst. HR-TEM confirmed that catalysts prepared using CeO_2_ sourced from Alfa Aesar were composed of cationic Pt species and performed comparatively poorly in this reaction. On the contrary, CeO_2_ that was produced in-house through the thermal decomposition of cerium (III) nitrate hexahydrate was much more active. Of these, the catalyst prepared using hexachloroplatinic acid (1% Pt/CeO_2_-DE-H-300) was more effective than the analogous catalyst prepared with tetraammineplatinum (II) nitrate (1% Pt/CeO_2_-DE-N-300). Through extensive characterization of these two catalysts, it was possible to determine the origin of the enhanced performance exhibited by the 1% Pt/CeO_2_-DE-H-300 catalyst. Its higher activity arises from the abundance of low-coordinate Pt species, which are present in the form of linear Pt species. Theoretical calculations determined that these features were also more effective at alcohol dehydrogenation, which is the RDS of the process. The enhanced DCPA selectivity exhibited by this catalyst can be attributed to the increased medium-strength basic sites, which are facilitated by the presence of residual Cl in the catalyst. These sites are less effective at promoting aldol condensation reactions, which are the primary competing reaction in the process. This study highlights how simple changes to catalyst preparation methods can dramatically influence the materials’ physicochemical properties and, consequentially, its catalytic performance. It also provides a simple but highly effective method of synthesizing Pt/CeO_2_ catalysts, which is highly effective in transfer amination reactions.

## Supporting Information Available

Testing data (time online plots), characterization data (STEM, SEM–EDX, XPS, in situ CO DRIFTS, ICP–MS, and CO chemisorption), and DFT calculations (PDF)
