# Supplementary material for: Uncovering Structure–Activity Relationships in Pt/CeO2 Catalysts for Hydrogen-Borrowing Amination
Source: ACS Catal. 2023 Jan 5;13(2):1207–20. doi: 10.1021/acscatal.2c04347 (PMC9872813; doi:10.1021/acscatal.2c04347)
Supplement: Supplementary file 1 — cs2c04347_si_001.pdf [file cs2c04347_si_001.pdf]

Electronic supplementary information for:

Uncovering structure-activity relationships in Pt/CeO_2 catalysts for hydrogen-borrowing amination

Tao Tong[1,2⊥], Mark Douthwaite[1⊥*], Lu Chen[2⊥], Rebecca Engel[1], Matthew B. Conway[1], Wanjun Guo[2], Xin-Ping Wu[2], Xue-Qing Gong[2*], Yanqin Wang[2*], David J. Morgan[1], Thomas Davies[1], Chris Kiely[3], Liwei Chen[4], Xi Liu[4*], Graham J. Hutchings[1*]

Table of Contents for electronic supplementary information

Item ID	Description	Page
Figure S1	Time courses of hydrogen-borrowing amination reaction over the 1%Pt/CeO_2-DE-H-300 and 1%Pt/CeO_2-DE-N-300 catalysts	S2
Figure S2	AC-STEM images of 1%Pt/CeO_2-DE-H catalysts reduced at 250, 300 and 350 ^oC.	S3
Figure S3	Low and high magnification STEM micrographs for the 1% Pt/CeO_2-DE-H-300, 1% Pt/CeO_2-DE-N-300 and 1% Pt/CeO_2-AA-H-300 catalysts	S3
Figure S4	CO-adsorption DRIFTS spectra for the Pt/CeO_2-AA-H-300 and Pt/CeO_2-Sigma-H-300 catalysts, respectively.	S4
Figure S5	XPS spectra of the Pt 4f orbit in unreduced 1%Pt/CeO_2-DE-H and 1%Pt/CeO_2-DE-N catalysts and Cl(2p) spectra for a series of fresh and reduced catalysts made with chloride and nitrate precursors showing chlorine states at 197.8 eV and 198.5 eV.	S4
Figure S6	Optimized structures for the initial state (IS), transition states (TS1 and TS2), and intermediates (IM1 and IM2) in the dehydrogenation of cyclopentanol to cyclopentaldehyde on model I.	S5
Figure S7	Optimized structures the initial state (IS), transition states (TS1 and TS2), and intermediates (IM1 and IM2) in the dehydrogenation of cyclopentanol to cyclopentaldehyde on model II.	S5
Figure S8	Optimized structures for the initial state (IS), transition states (TS1 and TS2), and intermediates (IM1 and IM2) in the dehydrogenation of cyclopentanol to cyclopentaldehyde on model III.	S6
Figure S9	STEM analysis of the Pt/CeO_2-DE-H-300 sample after a standard hydrogen borrowing amination reaction in the presence of CPA and CPL at 140 °C	S6
Table S1	Textural properties of 1%Pt/CeO_2 catalysts	S7
Table S2	EDX results of different 1%Pt/CeO_2-H catalysts	S7
Table S3	Calculated Bader charges (e) and average charges (e) of the Pt atoms in the models I, II, and III.	S8

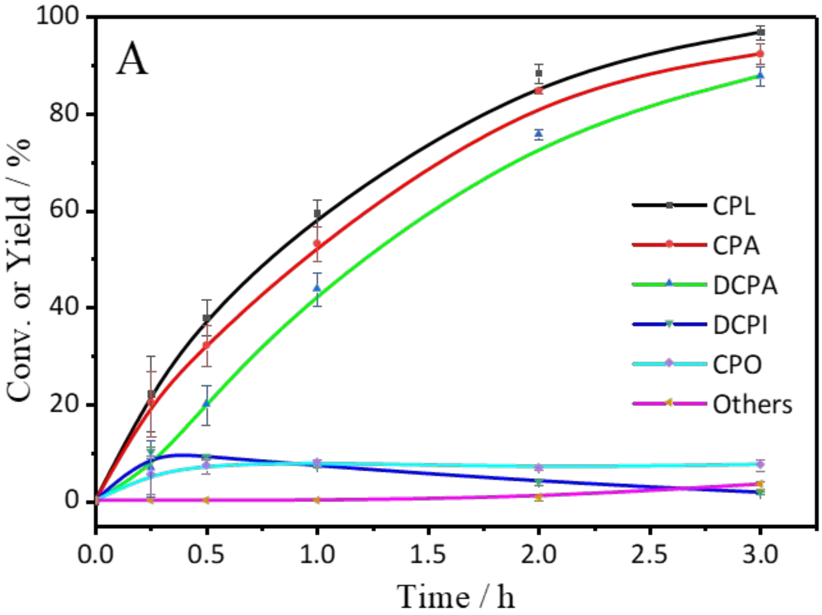

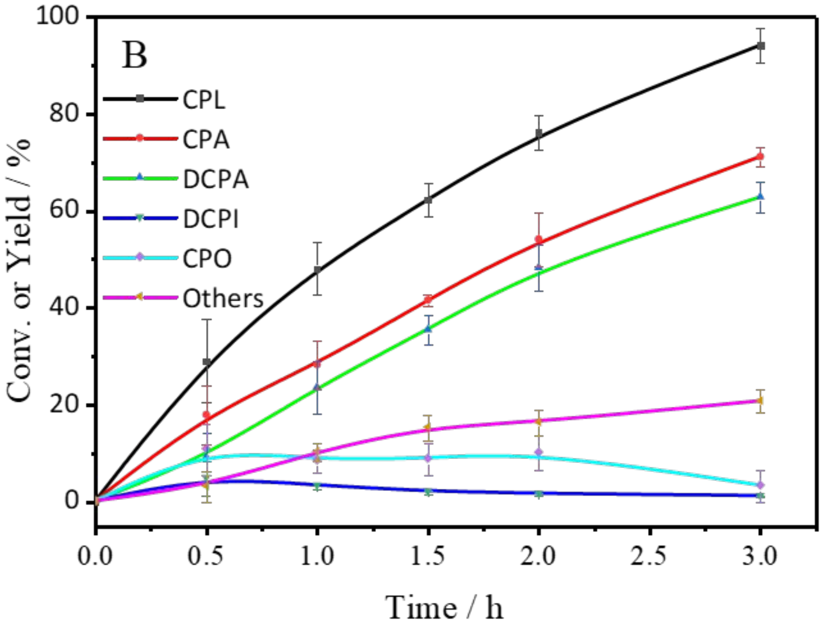

Figure S1. Time courses of hydrogen-borrowing amination reaction over (A) 1%Pt/CeO_2-DE-H-300; (B)1%Pt/CeO_2-DE-N-300 (Conditions: 2 mmol cyclopentanol, 2 mmol cyclopentylamine, 81.3 mmol *p*-Xylene (solvent), 140 ^{o}C, 50 mg catalysts, 1.54 mmol decane (Int.S), 800 rpm, 2 bar N_2).

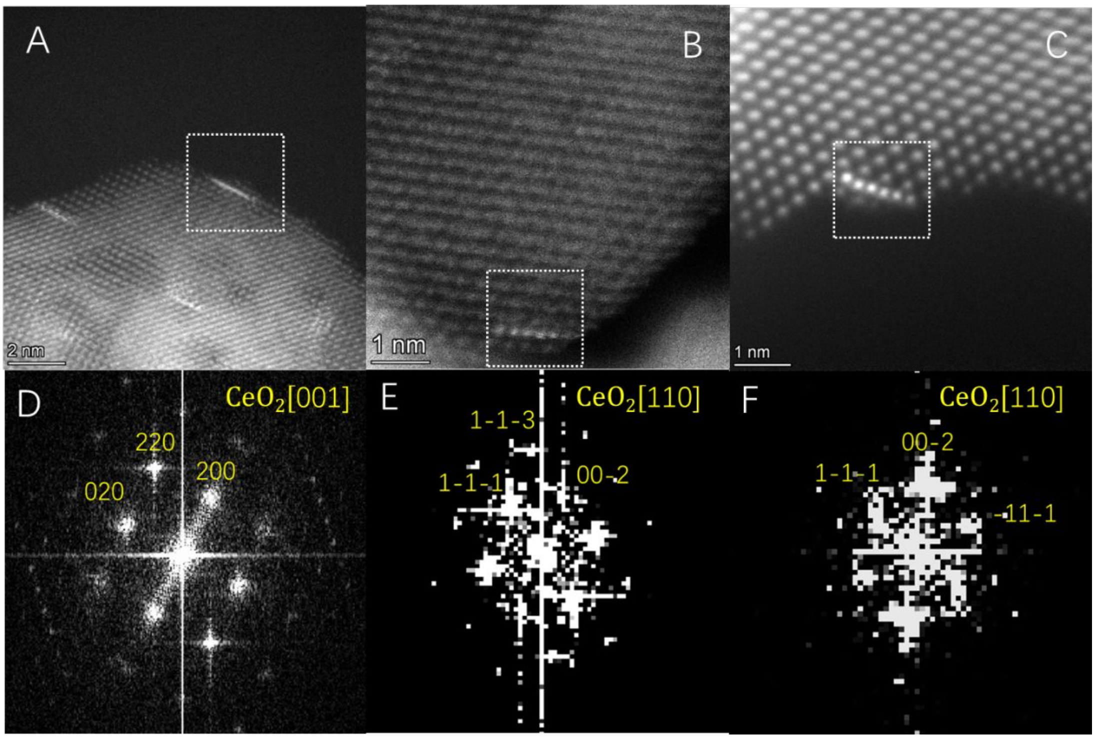

Figure S2. AC-STEM images of 1%Pt/CeO_2-DE-H catalysts reduced at (A) 250 ^oC; (B) 300 ^oC; (C) 350 ^oC. Selected area FFT was used to determine the CeO_2 orientations and preferable location of the linear Pt species.

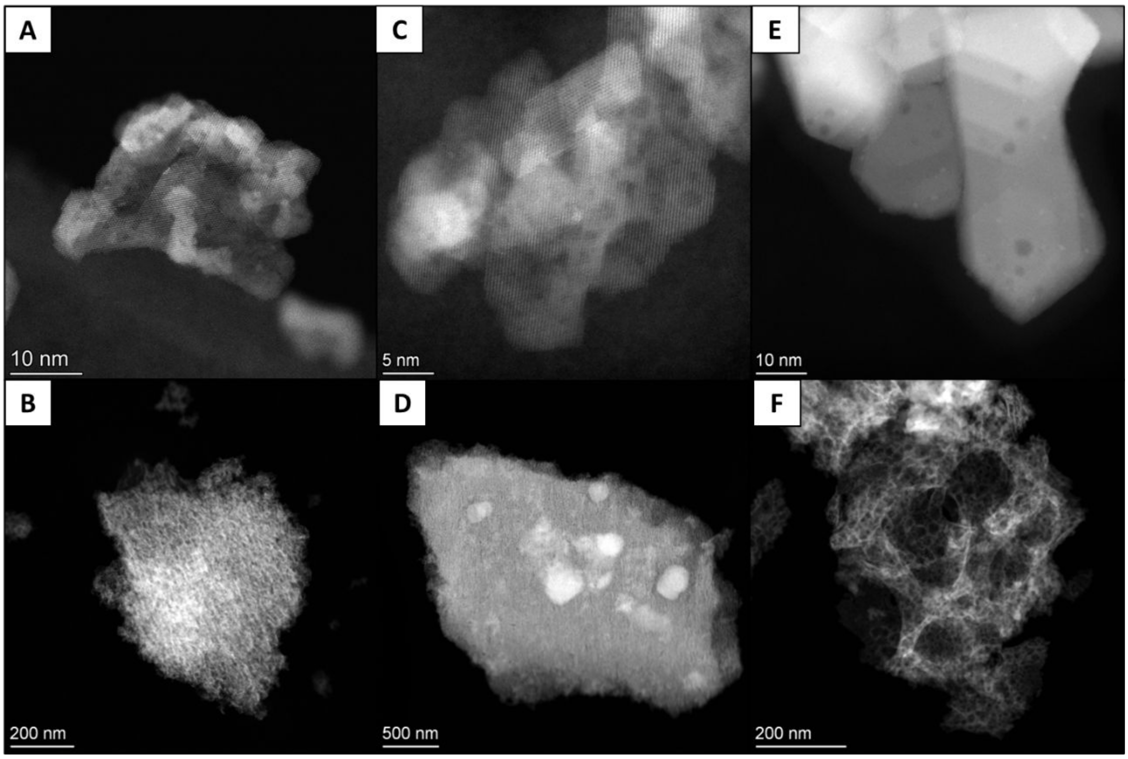

Figure S3. Low and high magnification STEM micrographs for the 1 *wt.*% Pt/CeO_2-DE-H-300 (**A** and **B**), 1 *wt.*% Pt/CeO_2-DE-N-300 (**C** and **D**) and the 1 wt.% Pt/CeO_2-AA-H-300 (**E** and **F**).

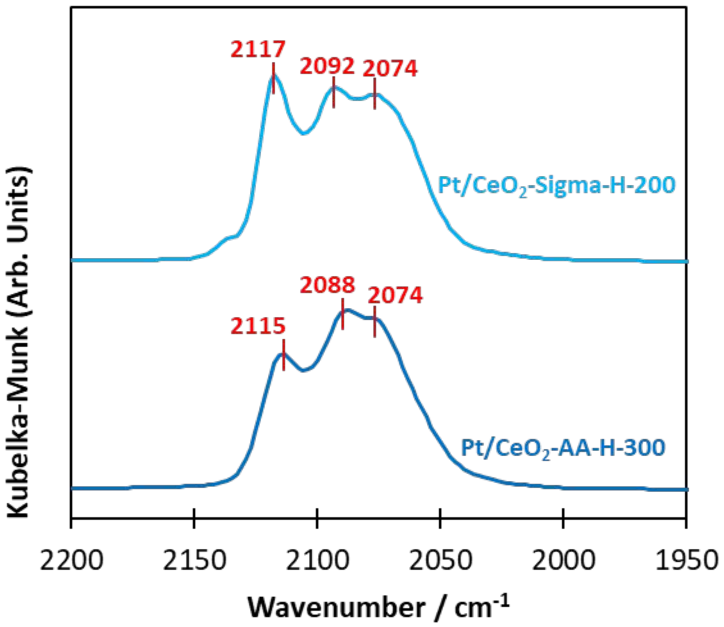

Figure S4. CO-adsorption DRIFTS spectra for the Pt/CeO_2-AA-H-300 and Pt/CeO_2-Sigma-H-300 catalysts, respectively.

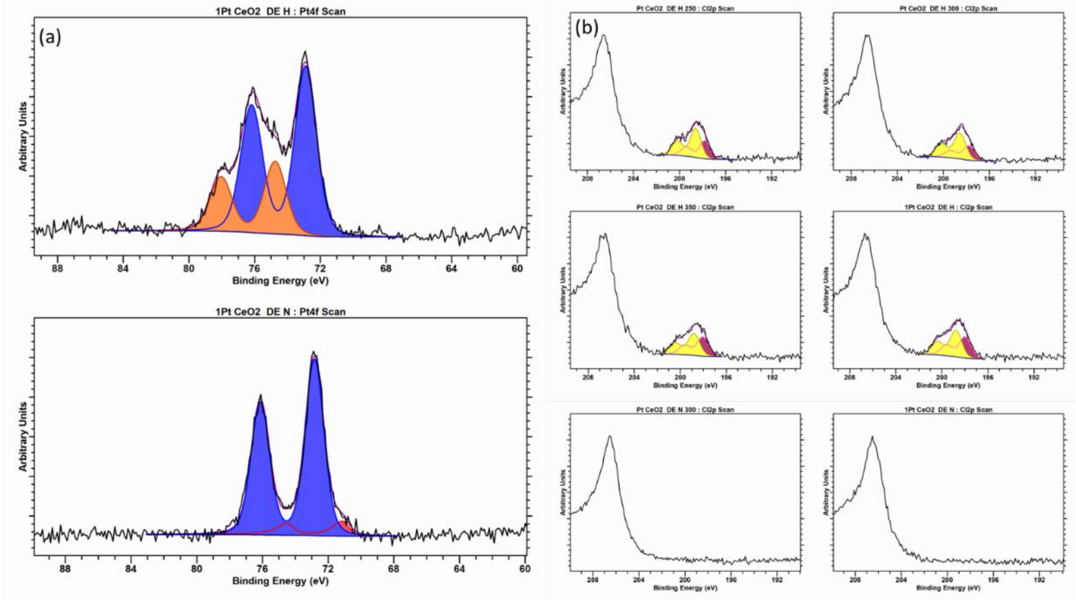

Figure S5. XPS spectra of (a) Pt 4f orbit of unreduced 1%Pt/CeO_2-DE-H and 1%Pt/CeO_2-DE-N samples. Red = Pt(0), Blue = Pt(II), $PtCl_x$/$Pt(OH)_2$ and orange = Pt(IV) as in $PtCl_4$ and (b) Cl(2p) spectra for a series of fresh and reduced catalysts made with chloride and nitrate precursors showing x2 chlorine states at 197.8 eV and 198.5 eV.

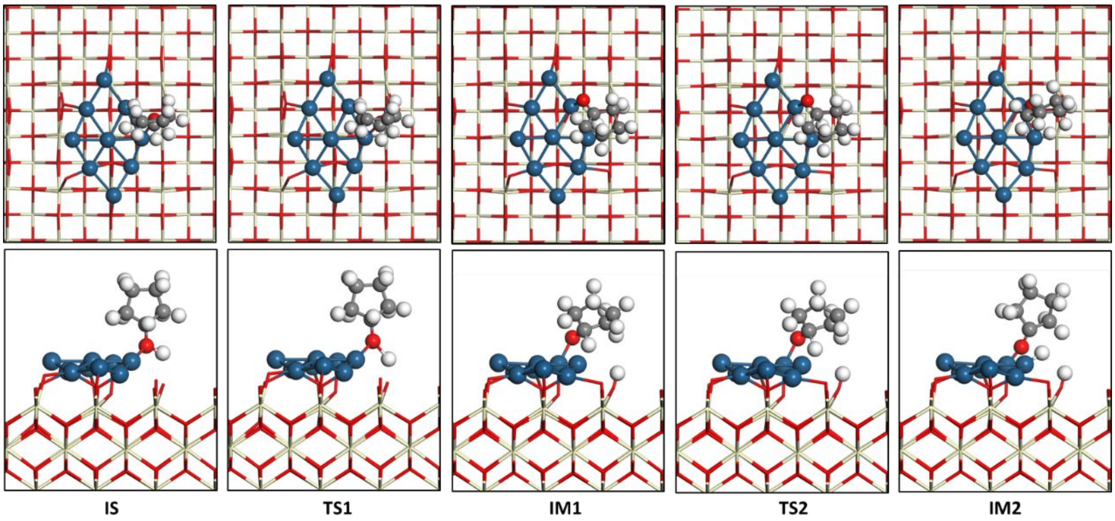

Figure S6. Optimized structures (top panel, top view; bottom panel, side view) for the initial state (IS), transition states (TS1 and TS2), and intermediates (IM1 and IM2) in the dehydrogenation of cyclopentanol to cyclopentaldehyde on the model I.

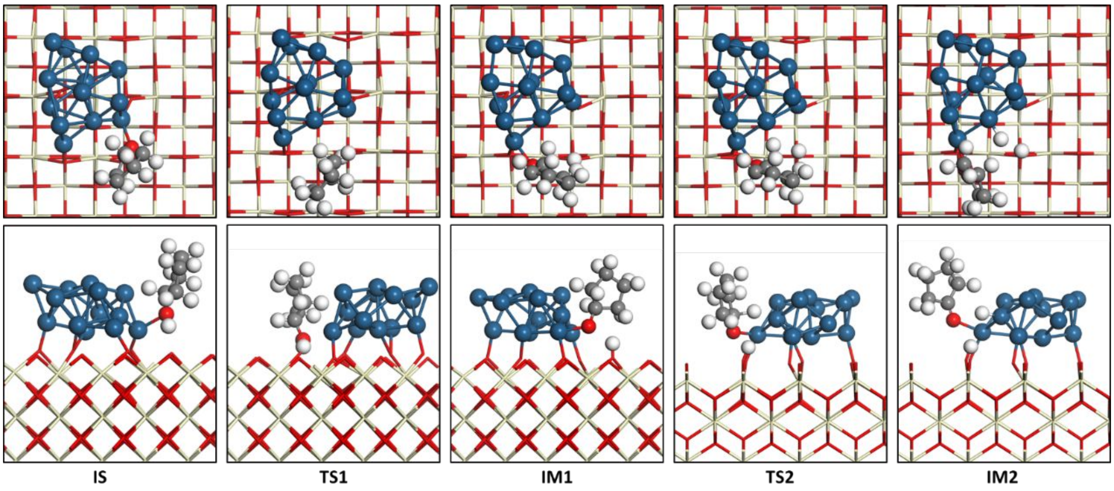

Figure S7. Optimized structures (top panel, top view; bottom panel, side view) for the initial state (IS), transition states (TS1 and TS2), and intermediates (IM1 and IM2) in the dehydrogenation of cyclopentanol to cyclopentaldehyde on the model II.

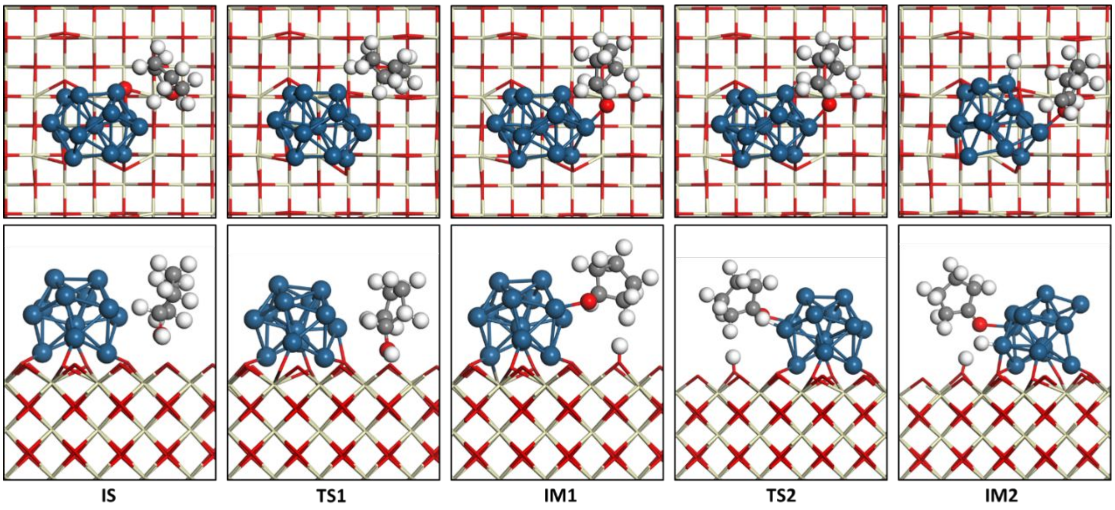

Figure S8. Optimized structures (top panel, top view; bottom panel, side view) for the initial state (IS), transition states (TS1 and TS2), and intermediates (IM1 and IM2) in the dehydrogenation of cyclopentanol to cyclopentaldehyde on the model III.

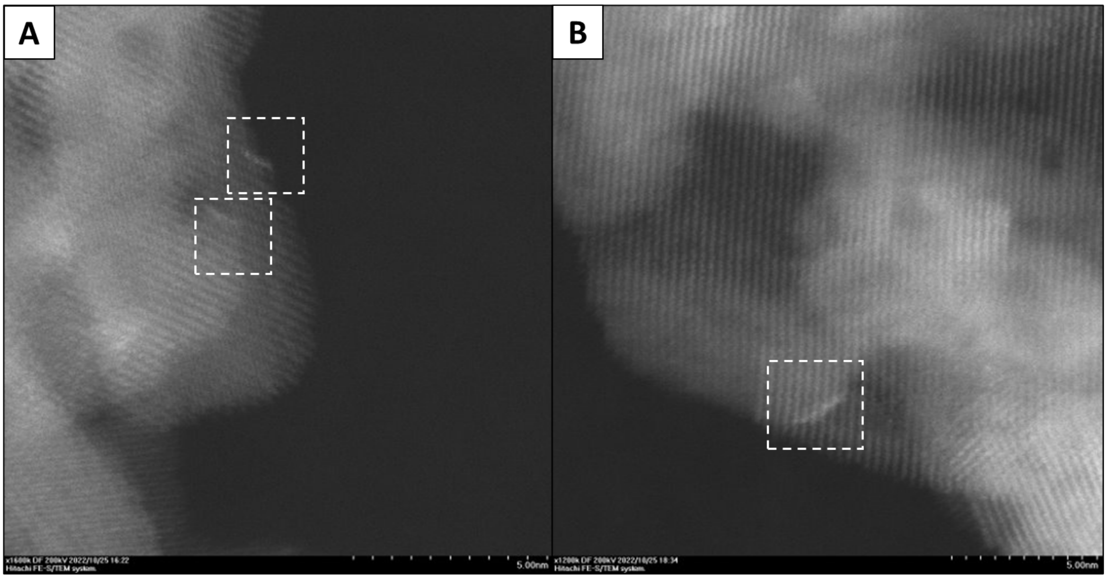

Figure S9. STEM analysis of the Pt/CeO_2-DE-H-300 sample after a standard hydrogen borrowing amination reaction in the presence of CPA and CPL at 140 °C after (A) 0.5 and (B) 2 hours of reaction, respectively. Linear Pt species are highlighted with white squares.

Table S1. Textural properties of the Pt/CeO_2 catalysts

Catalyst	Surface Area[a] (m^2 / g)	Time (h)	Conversion (%)	Pt Loading[b] (wt.%)	CO uptake[c] (mmol/g)	TOF (h^{-1})
Pt/CeO_2-DE-H-300	101	0.25	22.2	0.88	0.00947	3751
Pt/CeO_2-DE-N-300	66	0.50	29.0	0.82	0.00848	2736
Pt/CeO_2-AA-H-300	43	3.00	38.9	0.85	0.0214	242
Pt/CeO_2-Sigma-H-200	101	16.00	9.0	0.85	0.0174	13

a. Measured with N_2 Physisorption

b. Measured by ICP-OES

c. Measured by CO-Pulse chemisorption at 195 K

NOTE: CO adsorption on Pt is considered to be stoichiometric.

Table S2. SEM-EDS of the different Pt/CeO_2-H catalysts.

Catalyst	Average Pt (wt.%/atom.%)	Average Cl (wt.%/atom.%)
1%Pt/CeO_2-DE-H-300	0.95/0.23	1.12/1.5
1%Pt/CeO_2-AA-H-300	0.82/0.21	0.84/1.18
1%Pt/CeO_2-Sigma-H-300	0.96/0.24	1.07/1.48

Table S3. Calculated Bader charges (e) and average charges (e) of the Pt atoms in the models I, II, and III.

	model I	model II	model III
Pt_1	0.144[a]	-0.110	0.013
Pt_2	0.403[a]	0.079	0.014
Pt_3	0.483[a]	0.037	-0.029
Pt_4	-0.226[a]	0.018	-0.031
Pt_5	-0.047[a]	-0.081	-0.022
Pt_6	0.042[a]	-0.011	0.018
Pt_7	0.455[a]	0.057[a]	0.019
Pt_8	0.378[a]	0.341[a]	-0.021
Pt_9	0.115[a]	0.099[a]	0.149[a]
Pt_{10}	-	0.108[a]	0.126[a]
Pt_{11}	-	0.326[a]	0.122[a]
Pt_{12}	-	0.198[a]	-0.192[a]
Pt_{13}	-	0.161[a]	-0.210[a]
Average	0.194	0.094	-0.003

[a] charge of interfacial Pt
